# Biomarkers and prognostic factors of PD-1/PD-L1 inhibitor-based therapy in patients with advanced hepatocellular carcinoma

**DOI:** 10.1186/s40364-023-00535-z

**Published:** 2024-02-14

**Authors:** Nan Zhang, Xu Yang, Mingjian Piao, Ziyu Xun, Yunchao Wang, Cong Ning, Xinmu Zhang, Longhao Zhang, Yanyu Wang, Shanshan Wang, Jiashuo Chao, Zhenhui Lu, Xiaobo Yang, Hanping Wang, Haitao Zhao

**Affiliations:** 1 Department of Liver Surgery, State Key Laboratory of Complex Severe and Rare Diseases, Peking Union Medical College Hospital, Chinese Academy of Medical Sciences and Peking Union Medical College, No.1 Shuaifuyuan, Wangfujing, Beijing, 100730 China; 2 Department of Breast Surgery, Peking Union Medical College Hospital, Chinese Academy of Medical Sciences and Peking Union Medical College, No. 1 Shuaifuyuan, Beijing, 100730 China; 3 Hepatobiliary and Pancreatic Surgery, Shenzhen Qianhai Shekou Free Trade Zone Hospital, No.36 Industrial 8 Road, Nanshan District, Shenzhen City, Guangdong province China; 4 Division of Pulmonary and Critical Care Medicine, State Key Laboratory of Severe and Rare Diseases, Peking Union Medical College Hospital, Chinese Academy of Medical Sciences & Peking Union Medical College, Beijing, China

**Keywords:** Hepatocellular carcinoma, Programmed death-1, Programmed death ligand 1, Immune checkpoint inhibitors, Biomarker

## Abstract

Systemic therapies using programmed death-1 (PD-1) and programmed death ligand 1 (PD-L1) inhibitors have demonstrated commendable efficacy in some patients with advanced hepatocellular carcinoma (HCC); however, other individuals do not respond favorably. Hence, identifying the biomarkers, the prognostic factors, and their underlying mechanisms is crucial. In this review, we summarized the latest advancements in this field. Within the tumor microenvironment, PD-L1 expression is commonly utilized to predict response. Moreover, the characteristics of tumor-infiltrating lymphocytes are associated with the effectiveness of immunotherapy. Preclinical studies have identified stimulatory dendritic cells, conventional dendritic cells, and macrophages as potential biomarkers. The emergence of single-cell sequencing and spatial transcriptomics has provided invaluable insights into tumor heterogeneity through the lens of single-cell profiling and spatial distribution. With the widespread adoption of next-generation sequencing, certain genomic characteristics, including tumor mutational burden, copy number alterations, specific genes (TP53, CTNNB1, and GZMB), and signaling pathways (WNT/β-catenin) have been found to correlate with prognosis. Furthermore, clinical features such as tumor size, number, and metastasis status have demonstrated prognostic value. Notably, common indicators such as the Child-Pugh score and Eastern Cooperative Oncology Group score, which are used in patients with liver diseases, have shown potential. Similarly, commonly employed laboratory parameters such as baseline transforming growth factor beta, lactate dehydrogenase, dynamic changes in alpha-fetoprotein (AFP) and abnormal prothrombin, CRAFITY score (composed of C-reactive protein and AFP), and immune adverse events have been identified as predictive biomarkers. Novel imaging techniques such as EOB-MRI and PET/CT employing innovative tracers also have potential. Moreover, liquid biopsy has gained widespread use in biomarker studies owing to its non-invasive, convenient, and highly reproducible nature, as well as its dynamic monitoring capabilities. Research on the gut microbiome, including its composition, dynamic changes, and metabolomic analysis, has gained considerable attention. Efficient biomarker discovery relies on continuous updating of treatment strategies. Next, we summarized recent advancements in clinical research on HCC immunotherapy and provided an overview of ongoing clinical trials for contributing to the understanding and improvement of HCC immunotherapy.

**Supplementary Information:**

The online version contains supplementary material available at 10.1186/s40364-023-00535-z.

## Background

Cancer-related deaths due to hepatocellular carcinoma (HCC) rank fourth worldwide, presenting an abysmal outlook. Due to its insidious onset and asymptomatic nature, most patients are not diagnosed until later stages [1–3].

Immunotherapy is supported by the immune tolerance of the liver and the predominantly immunosuppressed microenvironment of HCC [4]. Since 2017, programmed death-1 (PD-1) inhibitors have been approved as second-line therapies for advanced hepatocellular carcinoma (aHCC). Although an encouraging response rate has been observed [5, 6], they have been unsuccessful due to insufficient statistical significance in subsequent phase III randomized clinical trials (RCTs) [7, 8]. Combination therapy is developing rapidly. Basic research has shown that the PD-1 inhibitor combined with tyrosine kinase inhibitors (TKIs) or anti-vascular endothelial growth factor (VEGF) antibody can enhance antitumor efficacy by increasing lymphocyte infiltration, weakening the immunosuppressive state, and promoting the normalization of blood vessels [9–11]. In clinical studies, the combination of atezolizumab, a programmed death ligand 1 (PD-L1) inhibitor, and bevacizumab, an anti-VEGF inhibitor, significantly prolonged progression-free survival (PFS) and overall survival (OS) compared with the classic treatment; thus, this combined treatment represents a new systemic treatment for HCC [12].

Good efficacy has been demonstrated in PD-1/PD-L1 inhibitor-based systemic therapy for aHCC; however, only a fraction of patients (15–40%) have benefited. Moreover, a significant percentage of patients who undergo treatment encounter disease progression (approximately 20–30%). Identifying biomarkers and prognostic factors for immunotherapy efficacy and their underlying mechanisms is crucial for patient selection, stratified management, and future related clinical research. Therefore, this review focuses mainly on research progress on biomarkers and prognostic factors of aHCC.

**Table 1:** The PD-L1 expression as the biomarker in the advanced hepatocellular carcinoma clinical trials of immunotherapy

Clinical Trial (Author, year) Reference	Regimen	Study design; number; line	PD-L1 positive vs. negative number	PD-L1 positive criteria	Outcomes (positive vs. negative)
CheckMate 040 (El-Khoueiry et al., 2017) [5]	Nivolumab	Interventional; N = 218; 1st and more (Sorafenib progressor or Sorafenib untreated or intolerant)	Escalation phase: 11 vs. 33; Expansion phase: 34 vs. 140	(Dako 28 − 8) TC ≥ 1%	Escalation phase: ORR (27% vs. 12%) Expansion phase: ORR (26% vs. 19%)
CheckMate 040 (Yau et al., 2020) [17]	Nivolumab plus ipilimumab	Interventional; N = 145^†^; 2nd	Arm A: 10 vs. 39; Arm B:10 vs. 38; Arm C: 8 vs. 40	(Dako 28 − 8) TC ≥ 1%	ORR (Arm A:30% vs. 31%; Arm B:30% vs. 32%; Arm C:50% vs. 28%)
Check Mate 459 (Yau et al., 2022) [14]	Nivolumab	Interventional; N = 366; 1st	71 vs. 295	(Dako 28 − 8) TC ≥ 1%;	ORR (28% vs. 12%) Median PFS (3.8 vs. 3.6 months) Median OS (16.1 vs. 16.7 months)
Keynote-224 (Zhu et al., 2018) [6]	Pembrolizumab	Interventional; N = 52; 2nd	CPS: 22 vs. 30	(Dako 22C3) CPS ≥ 1	ORR (32% vs. 20%)
			TPS: 7 vs. 45	(Dako 22C3) TPS ≥ 1%	ORR (43% vs. 22%)
GO30140 (Lee et al., 2020) [15]	Atezolizumab plus bevacizumab	Interventional; N = 86; 1st	1% cutoff: 61 vs. 25; 5% cutoff: 37 vs. 49; 10% cutoff: 30 vs. 56	(Ventana SP263) TC or IC ≥ 1%	ORR (41% vs. 28%)
				(Ventana SP263) TC or IC ≥ 5%	ORR (41% vs. 31%)
				(Ventana SP263) TC or IC ≥ 10%	ORR (50% vs. 30%)
NCT02989922 (Qin et al., 2020) [16]	Camrelizumab	Interventional; N = 30; 2nd	11 vs. 19	(Ventana SP142) TPS ≥ 1%	ORR (36% vs. 11%)
NCT03389126 (Lee et al., 2020) [18]	Avelumab	Interventional; N = 27; 2nd	22C3:6 vs. 21; SP263:8 vs. 19; SP142: 14 vs. 13; E1L3N: 10 vs. 17; PD-1 positive: 11 vs. 16	(Dako 22C3) TPS ≥ 1%	ORR (0.0% vs. 14.3%, P = 1.00); DCR (50.0% vs. 85.7%, P = 0.10)
				(Ventana SP263) TPS ≥ 1%	ORR (12.5% vs. 10.5%), P = 1.00); DCR (75.0% vs. 78.9%, P = 1.00)
				(Ventana SP142) IC ≥ 1%	ORR (21.4% vs. 0.0%, P = 0.22); DCR (71.4% vs. 84.6%, P = 0.65)
				(Cell Signalling E1L3N) score ≥ 1	ORR (20.0% vs. 5.9%, P = 0.54); DCR (60.0% vs. 88.2%, P = 0.15)
				PD-1 positive	ORR (18.2% vs. 6.2%, P = 0.55); DCR (81.8% vs. 75%, P = 1.00)
Imbrave150 (Cheng et al., 2022) [107]	Atezolizumab + Bevacizumab vs. Sorafenib	Interventional; N = 135; 1st	86 vs. 49	(Ventana SP263) TC or IC ≥ 1%	Median OS (22.8 (17.0-NE) vs.19.9 (13.9-NE)); Median PFS (7.0 (5.6–9.9) vs. 6.7 (5.4–10.0)); ORR (36% vs. 27%)
HIMALAYA (Abou-Alfa et al., 2022) [19]	STRIDE vs. Sorafenib Durvalumab vs. Sorafenib	Interventional; N = 681; 1st	STRIDE group:148 vs. 189; Durvalumab group:154 vs. 190	(Ventana SP263) TAP ≥ 1%	9-month OS rate: 68.2% vs. 67.7% (STRIDE group) 9-month OS rate: 69.5% vs. 74.2% (Durvalumab group)

† Arm A: Nivolumab 1 mg/kg plus ipilimumab 3 mg/kg Q3W (4 doses) followed by nivolumab 240 mg intravenously Q2W; Arm B: Nivolumab 3 mg/kg plus ipilimumab 1 mg/kg Q3W (4 doses) followed by nivolumab 240 mg intravenously Q2W; Arm C: Nivolumab 3 mg/kg Q2W plus ipilimumab 1 mg/kg Q6W.

Abbreviations: CPS, combined positive score; DCR, disease control rate; HCC, hepatocellular carcinoma; IC, immune cell; ORR, objective response rate; OS, overall survival; PD-1, programmed death-1; PD-L1, programmed death ligand 1; PFS, progression-free survival; STRIDE, Single Tremelimumab Regular Interval Durvalumab; TAP, tumor area positivity; TC, tumor cell; TPS, tumor cell proportion score

## Biomarkers of hepatocellular carcinoma immunotherapy

### Tumor microenvironment

#### PD-L1 expression

Even though PD-L1 expression remains a topic of debate in immunotherapy [13], most studies still support it as a predictor of response and prognosis (details are summarized in Table 1). According to CheckMate 040, PD-L1 expression is an effective biomarker. With expression ≥ 1% as the cut-off value for defining PD-L1 positive expression, positive individuals had a more promising objective response rate (ORR) than negative individuals [5]. Comparable results were obtained in the subgroup analysis of CheckMate 459, where individuals treated with nivolumab who obtained PD-L1 ≥ 1% were prone to experience longer median OS than those who did not [14]. In the KEYNOTE-224 study, the combined positive score (CPS) ≥ 1 was found to be a better predictor of high ORR and PFS compared to the tumor proportion score (TPS ≥ 1%) [6]. The phase 1b study (GO30140) [15] and phase II study (NCT02989922, camrelizumab) [16] also determined that PD-L1 expression was positively related to higher ORR.

However, another sub-cohort of the CheckMate 040 study showed contradictory outcomes [17]. Patients who underwent avelumab treatment exhibited similar unsatisfactory outcomes [18]. In the recent HIMALAYA trial, no matter the expression status, the combination of tremelimumab and durvalumab was beneficial for all subgroups in comparison to sorafenib alone [19].

There are several limitations with PD-L1 expression. The observed between-run heterogeneity level in HCC samples is notable, and HCC is distinguished by the presence of an immune cell-rich cirrhosis microenvironment. Consequently, there is strong spatial and cellular heterogeneity for its expression, which could potentially impact its predictive capability. Furthermore, it is noteworthy that PD-L1 status is subject to change over time, and utilizing a static specimen to determine PD-L1 status may not provide an accurate representation of the status during treatment [20]. Addressing these issues, such as minimizing heterogeneity and dynamically detecting PD-L1 expression, will be a critical area of focus for future research.

#### Tumor-infiltrating lymphocytes (TILs)

Immune checkpoint inhibitors (ICIs), specifically PD-1/PD-L1 inhibitors, are believed to be effective in HCC by activating existing immune responses within the tumor [21]. As a result, the potential possibility of TILs as a biomarker was noticed.

It has been shown that the density of TILs and treatment response are correlated. The subgroup analysis of the Checkmate 040 study indicated that individuals who achieved complete response (CR) or partial response (PR) exhibited a higher CD3 + TILs frequency than those with stable disease (SD). Furthermore, an increase in CD3 and CD8 TILs represented a trend toward enhanced OS, albeit not statistically significant [22]. Among those treated with tremelimumab, responders had a higher mean infiltration of CD3 + and CD8 + TILs after two doses of treatment compared to non-responders [23]. CD38 + TILs have also been linked to a favorable response [24].

As determined by recent research, the spatial distribution of TILs within the TME can impact a patient’s prognosis. A higher ratio of lymphocytes to total cell count (RLTCC) in non-tumoral regions was found to be linked with prolonged OS (median OS of 45.7 vs. 18.6 months; P = 0.006) in segmented histopathology images [25]. Effector T cells exert their antitumor effects only when specific clones of T cells are activated and expanded. Evidence suggests that the clonal structure of T cells within the tumor or in the surrounding area could potentially predict the response to ICI treatment. A previous study using T-cell receptor sequencing revealed that patients with higher clonality and T-cell fractions in their tumors tend to respond better to ICI therapy [23, 26].

To assess the activity of TILs, a measure called the Cytolytic Activity Score (CYT) has been developed. It evaluates the level of anticancer immunity through gene expression rather than simply relying on the density of TILs, as indicated by immunohistochemistry assays [27, 28]. One of the key benefits of CYT is its widespread accessibility and capacity for consistent replication at a minimal expense. In The Cancer Genome Atlas Program Liver Hepatocellular Carcinoma (TCGA-LIHC) cohort study [29], it was discovered that CYT was not influenced by tumor mutational burden (TMB). Furthermore, the high CYT score group exhibited an increased level of TCR richness, BCR richness, and TCR diversity, along with the presence of immune cell infiltration. Consequently, high CYT scores were associated with improved immunity and longer survival for HCC patients [29].

#### Other immune microenvironment markers

The immune microenvironment of HCC tumors is an intricate network comprising malignant cells, various immune cell populations, cytokines, and the extracellular matrix. Critical roles are played by these components during tumor progression. Intratumoral stimulatory dendritic cells (SDCs) can stimulate T cells by cross-presenting tumor antigens. In mouse models, this activity is essential to induce anti-PD-1 responses [30]. Moreover, numerous conventional DC 1s (cDC1s) have been associated with positive prognosis in anti-PD-1 therapy because upon taking cancer antigens, they migrate to lymph nodes where prime CD8 + T cells concentrate [31]. A recent study has demonstrated a significant positive correlation between intratumoral CD38 + CD68 + macrophage density and ICI response. This effect is likely attributed to the rising secretion of interferon γ (IFN-γ) and associated cytokines by CD38hi macrophages [24]. In the GO30140 phase 1b trial, individuals who obtained an elevated degree of VEGF receptor 2, Treg, myeloid inflammation, and triggering receptors expressed on myeloid cells 1/MDSC signatures exhibited improved PFS when administered with atezolizumab and bevacizumab (Atezo/Bev) in comparison to those who received with atezolizumab alone [32]. Cui et al. used machine learning methods to create an immune index composed of 10 genes to better represent the tumor microenvironment (TME) and anticipate the effectiveness of immunotherapy [33].

Due to the diverse and highly heterogeneous immune cells involved in tumor development, studies at the single-cell level are necessary to fully understand the TME. Such studies would provide a systematic and detailed tumor immune atlas, which would be beneficial for immunotherapy and discovering effective biomarkers [34]. The application of single-cell sequencing has revealed a correlation between increased levels of tumor cell diversity and unfavorable response when employing ICIs [34]. Ma et al. presented a single-cell atlas of liver tumors that were administered immunotherapy. The results showed that it is possible to predict the status of tumor cells utilizing functional clonality and immune response by measuring SPP1 expression [35]. With advances in spatial transcriptome technology and the integration of single-cell sequencing, it is possible to analyze the gene expression profiles and complete spatial information of tissues [36]. Recently, a study combining spatial transcriptomics and single-cell sequencing showed that in post-treatment samples of individuals who did not benefit from immunotherapy, the mutual interaction between SPP1-positive macrophages and cancer-associated fibroblasts (CAFs) formed the tumor immune barrier (TIB). More specifically, SPP1-positive macrophages may contribute to immune suppression, while CAFs may be involved in producing extracellular matrix components. Together, they limit the immune cells’ ability to kill tumor cells. Additionally, targeting SPP1 was validated in animal models to disrupt TIB structure, leading to enhanced effectiveness of immunotherapy [37]. Furthermore, an analysis of tissue samples from a group of 15 individuals who underwent neoadjuvant therapy with cabozantinib and nivolumab revealed that patients who were resistant to immunotherapy lacked CAF-enriched pro-inflammatory signaling, B cells with high activity, and HCC–CAF interactions [38]. Currently, the comparison of differences between HCC before and after immunotherapy has not been fully researched, which may provide a better understanding of micro changes in patients during treatment. In addition, regional therapies combined with immunotherapy plus molecular targeted therapies have gradually been studied in practical applications with promising efficacy [39], and a series of clinical trials, such as LEAP-012 and DEMAND, are also being conducted [40, 41]. Single-cell sequencing and spatial transcriptome sequencing have the potential to comprehend minute changes under such treatments.

### Genomic characteristics as predictive factors

The adaptive immune system primarily targets tumor-associated antigens that are expressed on cancer cells. Nonsense single nucleotide mutations (nsSNVs), also known as TMB, may impact the expression of these antigens and thus affect ICI-based immunotherapy effectiveness. Research on the relationship between TMB and HCC prognosis is limited. Studies have shown that the cut-off value varies between cancer types, with one study suggesting seven Muts/Mb as the cut-off value for HCC [42]. However, verification in a larger cohort is required due to limitations in sample size. Additionally, the number of genes that should be included to define TMB status (genome-wide, targeted group, or expression-only mutations) and potentially high mutational burden in key driver genes to act as predictive biomarkers remain undetermined. Further research is necessary to establish uniform diagnostic criteria.

As a hallmark of cancer, chromosomal instability causes widespread and focal copy number alterations (CNAs). Distinct molecular, immunological, and clinical characteristics are the outcome of CNAs. Research has found that the burden of CNAs is significantly correlated with the molecular typing and immunophenotyping of HCC [43]. According to Bassaganyas and colleagues, high broad CNAs are linked to immune exclusion and proliferation, and the CNA broad score could predict ICI therapy response in HCC [44].

The TP53 gene has been linked to the immune environment in HCC. As compared to individuals with wild-type TP53, those possessing TP53 mutations exhibited a shorter OS and recurrence-free survival [45]. CTNNB1 is another gene of interest, and basic research determined its role in immune escape and anti-PD-1 resistance [46]. It may function as a biomarker of immune rejection in individuals with aHCC. A small cohort enrolled HCC patients treated with ICIs showed that poor prognosis was related to altered WNT/β-catenin signaling, showing decreased disease control rate (0% vs. 53%), shorter median PFS (2.0 vs. 7.4 months), and shorter median OS (9.1 vs. 15.2 months) [47]. Additional investigations are suggested to comprehend its underlying mechanism on immunotherapy resistance [47, 48]. The GO30140 study showed that high expression of immune genes (CD274) and effector T signaling genes (GZMB, PRF1, and CXCL9) was linked with highly satisfying outcomes, including ORR and PFS. On the contrary, high expression of Notch pathway activation genes was a negative predictor [49]. The Checkmate 040 study subgroup results revealed that better ORR and OS were linked with high expression of inflammatory gene signals (CD274, CD8A, LAG3, and STAT1) [22].

### Clinical features of tumors

#### Tumor burden

The macroscopic features of a tumor, such as its size and location, are more easily noticeable to a clinician than its microscopic features.

The size of a tumor is a crucial prognostic factor. A study of 33 nivolumab-treated patients found that those with tumors smaller than 5 cm (P = 0.034) and albumin-bilirubin (ALBI) scores of 1 (P = 0.040) had a better prognosis [50]. The results remained significant in a multivariate analysis for tumors smaller than 5 cm and ALBI scores of 1. Another study of 261 patients with HCC in Korea showed that those with small tumors (< 10 cm) had a high likelihood of responding to therapy (11.4% vs. 5.5%) and better PFS and OS (P < 0.05) [51]. Moreover, Huang et al. found that in cases of multifocal HCC, small lesions had strong immune infiltration and were responsive to PD-1 inhibitors [52]. By incorporating both the size and number of malignant lesions in the liver, the tumor burden score (TBS) was significantly related to the treatment response in an immunotherapy cohort enrolling 378 patients with aHCC, and a TBS less than eight was correlated with longer OS [53].

#### Involved organs

Organ-specific responses in HCC immunotherapy vary, and lung metastases are often a positive indicator for immunotherapy. Taiwanese researchers conducted a study on 75 patients with aHCC and discovered that the response of HCC located in different organs was significantly different, and the patients with lung metastases achieved the best ORR. Extrahepatic lesions were more easily controlled in patients with both intrahepatic and extrahepatic lesions [54]. Other studies have also reported a similar trend, with lung and lymph node metastases often indicating a good response to immunotherapy [50, 51, 55]. However, the reason for the organ-specific heterogeneous response remains unknown and may be related to factors such as small metastasis size and strong immune infiltration. Unlike targeted therapy, extrahepatic metastasis does not negatively impact the prognosis of immunotherapy, making it a favorable factor in HCC treatment.

### Host clinical features

The clinical characteristics of the patient (liver function, physical condition, changes in alpha-fetoprotein (AFP), etc.) remain very important in immunotherapy and represent stable prognostic factors.

#### Pretreatment factors

Among patients suffering from liver disease, Child-Pugh and ALBI scores are commonly applied to evaluate liver function. Its impact on the prognosis of immunotherapy has been demonstrated to be significant. Child-Pugh B scores were associated with shorter median OS (2.8 months) than Child-Pugh A scores (10.7 months) in a Korean study of 203 individuals (P < 0.01) [56]. Pinato et al. discovered that the ALBI score was an independent predictor, with a median OS of 22.5 months for an ALBI score of 1, 9.6 months for an ALBI score of 2, and 4.6 months for an ALBI score of 3 (P < 0.001) [57]. However, the impact on short-term response remains controversial.

Physical fitness, as measured by the Eastern Cooperative Oncology Group (ECOG) score, has also been found to be a potential factor in the prognosis of immunotherapy. A Taiwanese study found that in terms of PFS and OS, patients with an ECOG score of 0 significantly outperformed those with a score of 1 or greater [55]. On the contrary, for a group of 233 patients who received nivolumab, univariate analysis indicated that the ECOG score was a borderline predictor of survival (P = 0.05) but not in multivariate analysis [58]. Furthermore, research conducted in Taiwan, involving 45 individuals diagnosed with aHCC and undergoing nivolumab treatment, discovered that the patient-generated subjective global assessment (PG-SGA) score was also an independent predictor of treatment efficacy, with a PG-SGA score < 4, indicating a greater susceptibility to disease control [59].

A patient’s underlying medical condition also impacts immunotherapy. A meta-analysis that enrolled high-evidence RCTs determined that individuals with nonviral HCC cannot benefit from immunotherapy. However, individuals diagnosed with viral-related HCC may benefit from immunotherapy, as evidenced by a pooled hazard ratio of 0.64 (95% CI 0.5–0.83) for OS [60].

The CRAFITY score, which utilizes both serum AFP and C-reactive protein (CRP) levels, shows its potency as a prognostic tool in aHCC immunotherapy. In a multivariate analysis, baseline serum AFP levels ≥ 100 ng/mL and CRP levels ≥ 1 mg/dL were recognized as autonomous indicators [61]. Yang et al. recently applied the CRAFITY score to individuals treated with PD-1 inhibitors plus TKIs in China, with promising outcomes [62].

Studies have also shown that high baseline plasma levels of Transforming Growth Factor beta (≥ 200 pg/mL) [63] and elevated lactate dehydrogenase (LDH) [51] are significant risk factors for poor prognosis of HCC immunotherapy. The LIPI score, which consists of the pretreatment-derived neutrophil-lymphocyte ratio (dNLR) and LDH, has also been shown to predict PFS and OS [64]. An elevated baseline level of interleukin-6 (IL-6) has been recognized as a negative indicator for non-response to treatment with Atezo/Bev [65].

In the clinical diagnosis of HCC, imaging examinations are of utmost importance. Several studies suggest that imaging may be an effective non-invasive biomarker for immunotherapy in HCC. Research has revealed that gadolinium ethoxybenzyl diethylenetriamine pentaacetic acid (Gd-EOB-DTPA)-enhanced magnetic resonance imaging (EOB-MRI) can be applied as a valuable approach to identify β-catenin mutations [66]. By applying EOB-MRI, a small study of 18 patients receiving ICI monotherapy found that higher intensity of the nodule during the hepatobiliary phase was correlated with shorter PFS (2.7 months vs. 5.8 months, P = 0.007) [67]. An additional study of 35 Atezo/Bev-treated patients revealed that signal intensity in the hepatobiliary phase could somehow forecast treatment response [68]. The complex TME leads to increased liver stiffness, and immunotherapy response results in a decrease in viable tumor cells but an increase in immune content, which can impact the function of immune cells, causing alterations in stromal and fibrosis composition. Based on these findings, a small prospective cohort under the anti-PD-1 regimen showed the potential of magnetic resonance elastography (MRE) to predict the prognosis [69]. A subsequent study involving 25 patients with the same regimen demonstrated that an absence of capsular enhancement in MRI enhancement and increased stiffness measured by MRE are both associated with unsatisfactory outcomes (P < 0.001) [70]. Thus far, research on imaging biomarkers is mainly restricted to monotherapy, and their efficacy in dual regimens (anti-PD-1 plus TKIs or anti-PD-1 plus anti-cytotoxic T lymphocyte-associated antigen 4 [CTLA-4]) remains unclear. Due to the heterogeneity of imaging evaluations in different centers, future multicenter analyses are necessary.

Positron emission tomography-computed tomography (PET/CT) has been widely applied to clarify malignant lesions and assess the extent of metastasis. Some scientists have examined the potential of PET/CT to investigate biological indicators. Evidence suggests that ^18^ F-fluorodeoxyglucose (^18^ F-FDG) PET/CT can predict the prognosis of individuals who received immunotherapy combined with molecular targeted drugs. Wang et al. found that total lesion glycolysis was associated with the outcomes of immunotherapy [71], while another study found that Metabolic Tumor Volume was a more promising parameter to forecast immunotherapy effectiveness [72]. A predictive model constructed by combining clinical parameters (ECOG score, Child-Pugh score, and bone metastasis situation) was able to effectively distinguish patients based on their treatment benefit [72]. Additionally, dual-tracer development has attracted attention, with ^11^ C-acetate and ^18^ F-FDG PET/CT exhibiting the potential to differentiate those who are prone to benefit from TKIs or immunotherapy [73]. Another tracer, the ^68^Ga-labeled FAP inhibitor (^68^Ga-FAPI), was also utilized as a predictive tool. Wu et al. reported that the ^68^Ga-FAPI–avid tumor volume in baseline PET/CT was associated with unsatisfactory clinical benefits in the regimen of PD-1 inhibitors plus lenvatinib [74]. These findings suggest the potential of PET/CT as a valuable tool in immunotherapy.

#### Post-treatment factors

According to research conducted in Taiwan, prognoses were better when AFP levels declined within the first four weeks (early AFP changes) following systemic therapy. After considering other parameters, it remained an independent predictor of a better outcome [75]. Dynamic variation of prothrombin induced by vitamin K absence-II (PIVKA-II) was also associated with prognosis, with a > 50% reduction six weeks after the initial anti-PD-1 therapy indicating longer PFS and OS [76].

Hematological examinations play a key role as they are non-invasive and can offer dynamic monitoring. For individuals with Child-Pugh A, Dharmapuri et al. found those who experienced a lower neutrophil-lymphocyte ratio (NLR < 5) and platelet-to-lymphocyte ratio (PLR) after three doses of nivolumab were more likely to be the responders. In the multivariate analysis, survival was strongly linked to the NLR and PLR after treatment, and an integrated model that included both showed greater significance [77]. Another study conducted in Korea with 189 patients receiving nivolumab revealed that the development of hyperprogressive disease (HPD) on immunotherapy was found to be associated with an increased NLR ratio (> 4.125) (AUC = 0.844) as well as worse PFS and OS [78].

Immune-related adverse events (irAEs) are the consequence of elevated immune system activity stimulated by ICIs. It is hypothesized that irAEs and improved clinical outcomes may be associated due to their similar underlying immunological processes. An analysis of 168 patients with aHCC showed that multisystem involvement and severe irAEs could predict better treatment outcomes, with significantly improved median PFS and median OS [79]. Additionally, studies have shown specific site irAEs related to patient prognosis [80]. The site varies between different types of cancer [81], potentially owing to molecular mimicry that may exist between malignant and normal cells. Currently, there is no site-specific irAE linked with the prognosis of patients with HCC, although a trend was observed in dermatological and endocrine irAEs [79]. The use of irAEs as a biomarker for immunotherapy remains controversial [82]; further investigation is necessary to ascertain their efficacy.

### Liquid biopsy

Liquid biopsy of tumors mainly involves the analysis of circulating tumor DNA (ctDNA), circulating tumor cells (CTCs), and cell-free DNA (cfDNA). This method of biopsy is minimally invasive, convenient, and easily repeatable; thus, it has gained increasing recognition for its usefulness in the dynamic guidance of immunotherapy, detection of drug resistance, and assessment of prognosis [83].

PD-L1 + CTCs are considered an attractive target. For those treated with nivolumab or pembrolizumab, favorable outcomes were observed when PD-L1 + CTCs were present [84]. Additional investigation is required to validate its accuracy in the context of HCC. Another potential biomarker is ctDNA, which has been recognized as a possible indicator. ctDNA content fraction (CCF) was significantly correlated with clinical outcomes in a pan-cancer cohort [85]. A study that enrolled 48 patients with aHCC indicated higher baseline ctDNA was correlated with higher TMB, while decreases in ctDNA levels after treatment were linked with longer PFS [86]. Franses et al. also revealed a significant correlation between tissue TMB and blood TMB estimated by ctDNA [87]. In addition, a risk-scoring model based on cfDNA copy number variation (CNV) has been developed to forecast the clinical outcomes of hepatobiliary tumor patients receiving ICI therapy. The model has been tested in two separate ICI treatment groups, and it was found that individuals with lower CNV risk scores had better PFS and OS [88].

### Commensal microorganisms

Commensal microorganisms, collectively known as the microbiota, have been shown to impact human immune responses in both healthy and diseased conditions [89, 90]. For gut microbiota, studies have demonstrated that the diversity and makeup could influence immunotherapy response in both mice and humans [91]. In a study involving aHCC patients treated with immunotherapy, fecal samples demonstrating increased diversity in terms of taxa and high gene counts were associated with positive treatment response. Dynamic sampling also showed that the gut microbiome dynamic variation 3–6 weeks after initial therapy exhibited the potential capability to predict the durable clinical benefit from immunotherapy, which is valuable for early prediction [92]. Another study enrolled 65 hepatobiliary tumor patients who received anti-PD-1-based therapy and found those with a higher abundance of Lachnospiraceae bacterium-GAM79 and *Alistipes* sp. Marseille-P5997 achieved better survival benefits. Unfavorable results were linked to the greater prevalence of Veillonellaceae [93]. Another study also indicated the potential of its composition as a biomarker. Patients with abundant *Prevotella* 9 had significantly shorter OS, and those with abundant *Lachnoclostridium* presented significantly longer OS. Moreover, individuals with abundant *Lachnoclostridium* and reduced *Prevotella* 9 in feces had the best OS. Notably, bile acids regulated by gut microbiota were also partially associated with ORR [94]. However, current research on gut microbiota has certain limitations. Moreover, dynamic studies of gut microbiota or metabolites are currently inadequate. With the development of metabolomics, the combined study of microbiome and metabolome can be used as a bridge connecting microbiomes and phenotypes. Independent microbiome and phenotype data can be effectively combined to achieve a comprehensive analysis of the microbial-metabolism-host interaction mechanisms. It will provide vital information for determining the predictive role of gut microbiota.

In addition to the aforementioned biomarkers, technological advancements and the continuous evolution of therapeutic approaches have paved the way for ongoing prospective clinical studies aiming to elucidate biological markers for HCC treatment from various perspectives. Table 2 shows the current clinical trials investigating the biomarkers for HCC immunotherapy.

**Table 2:** The ongoing biomarkers exploration clinical trials in the immunotherapy of hepatocellular carcinoma

NCT number (Estimated Completion Date)	Regimen	Study design (Estimated number); country	Target population	Biomarker sample	Analysis methods	Focus
Mainly based on IHC						
NCT03753659 (June 2024)	Pembrolizumab + Local ablation (RFA, MWA, brachytherapy)	Interventional (N = 30); Germany	Histologically confirmed; Early-Stage HCC; ECOG PS of 0 or 1; Child-Pugh score of A.	Tumor tissue, blood	IHC, molecular analyses	Molecular biomarkers, immune cells, chemokines, invasion markers
NCT04443309 (August 2024)	Lenvatinib + Camrelizumab	Interventional (N = 53); China	Histologically, cytologically, or clinically confirmed; BCLC Stage B/C; ECOG PS of 0 or 1; Child-Pugh score of A.	Tumour samples, blood	IHC, RNA-sequencing	PD-L1 expression, CD8 + T cell
NCT04803994 (April 1, 2025)	Atezolizumab + Bevacizumab + TACE	Interventional (N = 434); Austria, Germany, Spain	Radiographic or pathologic diagnosis; intermediate-stage; Child-Pugh class A or B7; ECOG PS of 0 or 1.	Tumor, blood, stool samples	IHC, multi-omics analysis	Predictive biomarkers (tissue and circulating) for study endpoints, PD-L1 expression
Mainly based on NGS						
NCT04701060 (February 4, 2024)	Camrelizumab + Apatinib	Interventional (N = 30); China	Clinical diagnosis resectable HCC; Child-Pugh class A; ECOG PS of 0 or 1.	Tumour samples, blood	NGS, IHC	Genomic biomarkers (TMB, TNB, ITH, HLA subtype, HLA-LOH, etc.), TILs, PD-L1 expression
NCT04170556 (August 2024)	Regorafenib + Nivolumab	Interventional (N = 78); Spain	Histologically or clinically confirmed; Child-Pugh class A; ECOG PS of 0 or 1.	Serum and tissue	Not applicable	Serum and tissue marker characterization
NCT04134559 (January 1, 2025)	Pembrolizumab	Interventional (N = 18); United States	Histologically confirmed; relapsed/refractory pediatric HCC	Tumor samples, blood	IHC, DNA sequencing, liquid biopsy	Dynamic changes in infiltrating immune cells, cytokines, and ctDNA; genomic biomarkers
NCT04224636 (March 1, 2025)	Atezolizumab + Bevacizumab + TACE	Interventional (N = 106); Germany	Histologically confirmed; Child-Pugh class A or B7; ECOG PS of 0 or 1.	Tumor samples, blood, stool samples	Multi-omics analysis	Serum marker, cytokines, ctDNA, gut microbiome
NCT04145141 (December 31, 2025)	Immunotherapy	Observational (N = 500); United States	Histologically/ultrasound/imaging confirmed	Blood, urine, and stool samples or rectal swabs	Multi-omics analysis	Genomic, genetic, and epigenetic analysis
NCT05286320 (September 30, 2026)	Pembrolizumab + Lenvatinib + SBRT	Interventional (N = 27); Chinese Taiwan	Histologically or clinically confirmed; patients with PVTT (VP3, VP4); ECOG PS of 0.	Pre-treatment tumor samples, blood	Not applicable	Biomarkers for the response of portal vein tumor thrombosis, PFS, and OS
NCT04246177 (December 31, 2029)	Lenvatinib + Pembrolizumab + TACE	Interventional (N = 450); Global	Radiology, histology, or cytology confirmed; HCC localized to the liver and not amenable to curative treatment; ECOG PS of 0 or 1.	Tumor samples, blood	Multi-omics analysis	Genomic, metabolic, and proteomic biomarker
Mainly based on single-cell sequencing						
NCT05173298 (December 31, 2024)	Atezolizumab + Bevacizumab	Observational (N = 100); South Korea	Histologically, cytologically, or clinically confirmed; treatment-naïve	Tumour samples, blood	Tumor samples: H&E staining and IF staining. Blood samples: flow cytometry, ELISA, single-cell sequencing	Protein biomarker, gene-based biomarker
NCT03419481 (December 30, 2024)	Pembrolizumab	Interventional (N = 30); Hong Kong	Confirmed diagnosis of HCC; ECOG PS of 0 or 1	Baseline and post-treatment tumor samples (after two cycles of Pembrolizumab)	Single-cell sequencing, IHC	The serial change in cytokine profile, PD-L1 expression, TILs, the serial change in RNA expression of immune-related gene panel
Others						
NCT03864211 (May 30, 2023)	Thermal ablation + Toripalimab	Interventional (N = 145); China	Clinically confirmed; Child-Pugh class A/B; ECOG PS of 0 or 1.	Blood samples	Liquid biopsy	Dynamic changes in inflammatory biomarkers.
NCT05278195 (December 1, 2023)	Anti-PD-1/PD-L1 + VEGF/TKI + TACE	Observational (N = 300); China	Histologically, cytologically, or clinically confirmed; treatment-naïve	Imaging information	Radiomics artificial intelligence model	Imaging biomarkers
NCT05044676 (September 30, 2024)	Atezolizumab + Bevacizumab	Prospectively observational (N = 120); France	Advanced HCC with an indication of systemic therapy by Atezo/Bev; ECOG PS of 0 or 1.	Blood, tumor samples (tumoral and non-tumoral liver) with dynamic monitoring	Flow cytometry	Immune cells biomarker (the frequency and phenotype expression of CD226 on CD8 + T lymphocytes and NK cells); A predictive prognostic score from histological characteristics
NCT04368078 (April 2025)	Lenvatinib + Toripalimab	Interventional (N = 76); China	Histologically, cytologically, or clinically confirmed; BCLC Stage B/C; ECOG PS of 0 or 1; Child-Pugh score of A.	Tumor samples, blood, stool samples	Multi-omics analysis	Potential biomarkers of treatment response
NCT04522544 (September 30, 2025)	Durvalumab + Tremelimumab + Y-90 SIRT/ TACE	Interventional (N = 84); Germany	Histologically confirmed; Child-Pugh class A; ECOG PS of 0 or 1.	Tumor tissue and blood samples	IHC, ELISA, liquid biopsy	PD-L1 expression, infiltrating immune cells, chemokines, invasion markers, circulating nucleic acids, and tumor-specific transcripts
NCT03475953 (December 31, 2025)	Regorafenib + Avelumab	Interventional (N = 747); France	Histologically confirmed; ECOG PS of 0 or 1; Child-Pugh A.	Tumor tissue and blood samples	Liquid biopsy, IHC, liquid chromatography-mass spectrometry	Predictive blood biomarkers analysis (cytokines levels, lymphocytes); Predictive tumor growth factor biomarkers; Predictive metabolomic analysis

Abbreviations: Azteo/Bev, atezolizumab and bevacizumab; BCLC, Barcelona clinic liver cancer; ctDNA, circulating tumor DNA; ECOG PS, ECOG Performance Status; ELISA, enzyme-linked immunosorbent assay; HCC, hepatocellular carcinoma; HLA, human leukocyte antigen; LOH, loss of heterozygosity; H&E, Hematoxylin & eosin; ITH, intra-tumor heterogeneity; IHC, immunohistochemical; IF, immunofluorescence; MWA, microwave ablation; NGS, Next-generation sequencing; OS, overall survival; PD-1, programmed death-1; PD-L1, programmed death ligand 1; PFS, progression-free survival; PVTT, portal vein tumor thrombosis; RFA, radiofrequency ablation; SBRT, stereotactic body radiation therapy; SIRT, selective internal radiation therapy; TILs, Tumor-infiltrating lymphocytes; TKI, tyrosine kinase inhibitor; TACE, transarterial chemoembolization; TMB, tumor mutational burden; TNB, tumor neoantigen burden; VEGF, vascular endothelial growth factor

## Relevant clinical studies of hepatocellular carcinoma immunotherapy

### Targeted therapy

Prior to the advent of immunotherapy, targeted therapies were crucial in the treatment of aHCC. The revolutionary SHARP trial in 2008 marked the beginning of the application of targeted therapies for the treatment of aHCC. Over the next decade, sorafenib has consistently remained the standard regimen, extending the median OS by 10.7 months [95]. However, in 2018, lenvatinib was shown to be comparable to sorafenib in the REFLECT trial. It demonstrated favorable safety and tolerability and was the second TKI approved for the first-line treatment of aHCC [96]. Subsequently, a study in a Chinese population showed that donafenib exhibited superior survival benefits than sorafenib [97]. Furthermore, several other TKIs have received approval as subsequent-line systemic therapies for aHCC. Regorafenib demonstrated promising efficacy in sorafenib-resistant patients, significantly improving median OS and ORR when compared with the placebo [98]. Similarly, cabozantinib was shown to improve median OS for sorafenib-resistant patients, although it did not extend the median PFS or ORR [99]. In the Chinese population, apatinib has exhibited benefits as a later-line therapy, significantly improving median PFS and median OS. Notably, this study included patients who had developed resistance to oxaliplatin-based chemotherapy [100]. In second-line treatment, ramucirumab, a VEGF receptor 2 inhibitor, has been proven to significantly improve survival in the population with AFP levels greater than 400 ng/mL [101].

### PD-1 inhibitor monotherapy

The CheckMate 040 study found that nivolumab can achieve a 20% ORR in aHCC [5]. Subsequently, a phase III RCT for first-line therapy, CheckMate 459, revealed that in contrast with sorafenib, nivolumab prolonged OS; moreover, the ORR (15% vs. 7%) and safety were more promising in the nivolumab group [102]. The KEYNOTE-224 study found that pembrolizumab could achieve an ORR of 17% as a second-line treatment of aHCC. After a 2.5-year follow-up, the ORR in the updated KEYNOTE-224 study reached 18.3%. The median PFS and median OS were 4.9 and 13.2 months, respectively [6, 103]. Soon afterward, in the phase III KEYNOTE-240 study, pembrolizumab extended OS by three months compared with the placebo [8, 104]. In KEYNOTE-394, which enrolled Asian patients, pembrolizumab significantly improved OS, PFS, and ORR [105]. Recently, RATIONALE-301 also showed that tislelizumab was non-inferior to sorafenib in OS for treatment-naïve individuals, showing a trend of prolonged OS and clinical survival benefit [106]. Based on the above RCTs, PD-1 antibodies are valuable in treating aHCC, but they cannot yet challenge conventional therapy.

### PD-1/PD-L1 inhibitors combined with anti-VEGF Drugs

The IMbrave150 study, the first successful phase III RCT, showed that Atezo/Bev markedly improved OS and PFS in treatment-naïve aHCC compared with sorafenib. Moreover, the safety of the treatment was established [12, 107]. This positive result has led to new treatment guidelines for aHCC, suggesting that immunotargeted therapy has strong efficacy and controllable safety. Recently, tiragolumab (a T cell immunoglobulin and ITIM domain [TIGIT] monoclonal antibody) plus Atezo/Bev has shown promise. In the phase Ib/II clinical trial, MORPHEUS-liver, this regimen showed higher ORR and longer PFS compared to the Atezo/Bev group (confirmed ORR: 42.5% vs. 11.1%, median PFS: 11.1 months vs. 4.2 months) [108]. Although including limited subjects, the study holds significant research implications and may represent a direction for future drug development and cancer treatment. Specifically, it highlights the importance of reshaping the TME and enhancing immune recognition to improve the effectiveness of immunotherapy.

The ORIENT-32 trial of sintilimab in combination with bevacizumab biosimilar (Sin/Bev) achieved an ORR of 25% in an early phase II study [109]. A subsequent phase III confirmatory study made clear that Sin/Bev greatly improved median OS and PFS in comparison to sorafenib [110].

### PD-1/PD-L1 inhibitors combined with TKIs

Several large phase III RCTs of PD-1/PD-L1 inhibitors bonded with TKIs have been conducted, but the efficacy of this regimen compared with TKI monotherapy is controversial. For the portfolio of camrelizumab plus apatinib, a phase II RCT showed that the ORR in the first-line cohort was 34.3% and 22.5% in the second-line cohort, showing good therapeutic effects [111]. The subsequent phase III CARES-310 trial showed that compared with sorafenib, camrelizumab plus rivoceranib (also referred to as apatinib) therapy led to a 48% reduction in the risk of disease progression (median PFS: 5.6 vs. 3.7 months) and a 38% reduction in the risk of death (median OS: 22.1 vs. 15.2 months) [112]. In the LEAP-002 trial, lenvatinib combined with pembrolizumab showed an improvement in PFS over lenvatinib monotherapy, although the results did not satisfy the validity threshold [113]. Similarly, cabozantinib plus atezolizumab offered improved PFS compared with sorafenib alone but without improving OS [114]. According to a phase II RCT conducted recently in naive-treatment patients, tislelizumab combined with lenvatinib achieved a 38.7% ORR and a 9.7-month median PFS [115]. Additional clinical trials are being conducted to assess the effectiveness of this regimen.

### PD-1/PD-L1 inhibitor combined with CTLA-4 inhibitor

In another CheckMate 040 sub-cohort, different doses of nivolumab combined with ipilimumab were used to treat sorafenib-resistant aHCC. Approximately 30% of the cases responded to this regimen with a median OS of 22.8 months [17]. The latest HIMALAYA trial showed excellent efficacy of the Single Tremelimumab Regular Interval Durvalumab (STRIDE) regimen. STRIDE significantly outperformed sorafenib in OS (median OS: 16.4 vs. 13.8 months). However, tumor responses were substantially better with sorafenib, which presented a 22-month median duration of response [19]. Additional details on these critical trials are summarized in Table 3.

**Table 3:** The critical trials of unresectable hepatocellular carcinoma immunotherapy

Clinical trial (Author, year) NCT number	Phase Line	Patient number (Treatment vs. comparator)	Target population		Treatment vs. Comparator	Key outcomes (RECIST v1.1 criteria)		≥ 3 grade AEs
**Targeted therapy**								
SHARP (Llovet et al., 2008) [95] NCT00105443	Phase 3 1st	N = 602 (299 vs. 303)	No previous systemic therapy; BCLC Stage B/C; Child-Pugh class A; ECOG PS of 0–2.		Sorafenib vs. Placebo	mOS: 10.7 vs. 7.9 months, HR = 0.69, 95% CI 0.55–0.87, p < 0.001; time to radiologic progression: 5.5 vs. 2.8 months, HR = 0.58; 95% CI 0.45–0.74; p < 0.001; DCR: 43% vs. 32%, p = 0.002		Grade 3 teAEs: 39% vs. 24%; Grade 4 teAEs: 6% vs. 8%
REFLECT (Kudo et al., 2018) [96] NCT01761266	Phase 3 1st	N = 954 (478 vs. 476)	BCLC Stage B/C; Child-Pugh class A; ECOG PS of 0 or 1.		Lenvatinib vs. Sorafenib	mOS: 13.6 vs. 12.3 months, HR = 0.92, 95% CI 0.79–1.06; mPFS: 7.3 vs. 3.6 months, HR = 0.65, 95% CI 0.56–0.77, p < 0·0001; ORR: 18.8% vs. 6.5%		Grade ≥ 3 trAEs: 57% vs. 49%; Serious trAEs: 18% vs. 10%
ZGDH3 (Qin et al., 2021) [97] NCT02645981	Phase 2/3 1st	N = 665 (333 vs. 332)	BCLC Stage B/C; Child-Pugh score of B7 or less; ECOG PS of 0 or 1.		Donafenib vs. Sorafenib	mOS: 12.1 vs. 10.3 months, HR = 0.831, 95% CI, 0.699–0.988, p = 0.0245; mPFS: 3.7 vs. 3.6 months, p = 0.0570; ORR: 4.6% vs. 2.7%		Grade ≥ 3 drug-related AEs: 38% vs. 50%; Serious drug-related AEs: 7% vs. 7%
RESORCE (Bruix et al., 2017) [98] NCT01774344	Phase 3 2nd	N = 573 (379 vs. 194)	Disease progressed after sorafenib treatment; BCLC Stage B/C; Child-Pugh class A; ECOG PS of 0 or 1.		Regorafenib vs. Placebo	mOS: 10.6 vs. 7.8 months, HR = 0.63, 95% CI 0.50–0.79, one-sided p < 0·0001; mPFS: 3.4 vs. 1.5 months, HR = 0.43, 95% CI 0.35–0.52, p < 0·0001; ORR: 7% vs. 3%, one-sided p = 0·0200		Grade 3 teAEs: 56% vs. 32%; Grade 4 teAEs: 11% vs. 7%
CELESTIAL (Abou-Alfa et al., 2018) [99] NCT01908426	Phase 3 2nd and 3rd	N = 707 (470 vs. 237)	BCLC Stage B/C; Child-Pugh class A; ECOG PS of 0 or 1.		Cabozantinib vs. Placebo	mOS: 10.2 vs. 8.0 months, HR = 0.76, 95% CI 0.63–0.92, p = 0.005; mPFS: 5.2 vs. 1.9 months, HR = 0.44, 95% CI 0.36–0.52, p < 0·001; ORR: 4% vs.<1%		Grade 3 any AEs: 58% vs. 34%; Grade 4 any AEs: 10% vs. 3%
REACH-2 (Zhu et al.,2019) [101] NCT02435433	Phase 3 2nd	N = 292 (197 vs. 95)	BCLC Stage B/C; Child-Pugh class A; ECOG PS of 0 or 1; AFP ≥ 400ng/mL		Ramucirumab vs. Placebo	mOS: 8.5 vs. 7.3 months, HR = 0.710, 95% CI 0.531–0.949, p = 0.0199; mPFS: 2.8 vs. 1.6 months, HR = 0.452, 95% CI 0.339–0.603, p < 0·0001		Any grade serious trAE: 11% vs. 5%
AHELP (Qin et al., 2021) [100] NCT02329860	Phase 3 2nd and more	N = 393 (261 vs. 132)	BCLC Stage B/C; Child-Pugh class A; ECOG PS of 0 or 1.		Apatinib vs. Placebo	mOS: 8.7 vs. 6.8 months, HR = 0.785, 95% CI 0.617–0.998, p = 0.048; mPFS: 4.5 vs. 1.9 months, HR = 0.471, 95% CI 0.369–0.601, p < 0·0001; ORR: 11% vs. 2%		Grade 3–4 trAEs:77% vs. 19%
**PD-1 inhibitor monotherapy**								
CheckMate 459 (Yau et al., 2019) [102] NCT02576509	Phase 3 1st	N = 743 (371 vs. 372)	No previous systemic therapy; Child-Pugh class A; ECOG PS of 0 or 1.		Nivolumab vs. Sorafenib	mOS: 16.4 vs.14.7 months, HR = 0.85, 95% CI 0.72–1.02, p = 0.075		Grade 3 trAEs: 18% vs. 47%; Grade 4 trAEs: 4% vs. 2%; Grade 3 serious trAEs: 7% vs. 7%; Grade 4 serious trAEs: 2% vs. <1%
KEYNOTE-240 (Finn et al., 2019) [8, 104] NCT02702401	Phase 3 2nd	N = 413 (278 vs. 135)	BCLC Stage B/C; Child-Pugh class A; ECOG PS of 0 or 1.		Pembrolizumab vs. Placebo	mOS: 13.9 vs. 10.6 months, HR = 0.771; 95% CI 0.617–0.964; mPFS: 3.0 vs. 2.8 months, HR = 0.718; 95% CI 0.571–0.903; ORR: 18.3% vs. 4.4%		Grade 3–4 trAEs:19.4% vs. 7.5%
KEYNOTE-394 (Qin et al., 2022) [105] NCT03062358	Phase 3 2nd	N = 453 (300 vs. 153)	Asian patients with confirmed aHCC and progression on or intolerance to Sorafenib or oxaliplatin-based chemotherapy.		Pembrolizumab vs. Placebo	mOS:14.6 vs. 13.0 months, HR = 0.79; 95% CI 0.63–0.99, p value = 0.0180; mPFS: 2.6 vs. 2.3 months, HR 0.74, 95% CI 0.60–0.92, p = 0.0032; ORR: 13.7% vs. 1.3%		Grade 3–5 trAEs: 14.4% vs. 5.9%
RATIONALE-301 (Qin et al., 2023) [106] NCT03412773	Phase 3 1st	N = 674 (342 vs. 332)	BCLC Stage B/C; Child-Pugh class A; ECOG PS of 0 or 1.		Tislelizumab vs. Sorafenib	mOS:15.9 vs. 14.1 months, one-sided p = 0.04; ORR: 14.3% vs. 5.4%		Grade ≥ 3 AEs: 48.2% vs. 65.4%
KEYNOTE-224 (Zhu et al., 2018) [6, 103] NCT02702414	Phase 2 2nd	N = 104	previously treated with Sorafenib; Child-Pugh class A; ECOG PS of 0 or 1.	Pembrolizumab		ORR: 18.3%; mPFS: 4.9 months; mOS: 13.2 months.		Grade 3 trAEs: 24%; Grade 4 trAEs: 1%;
CheckMate 040 (El-Khoueiry et al., 2017) [5] NCT01658878	Phase 1/2 1st and more	N = 262	aHCC with or without HCV or HBV infection. Previous sorafenib treatment was allowed.	Nivolumab		ORR: 20% (All patients); 23% (uninfected untreated/intolerant), 21% (uninfected progressor), 20% (HCV infected), 14% (HBV infected)		Dose-escalation phase: Grade 3–4 serious trAEs:17%(0.1 mg/kg), 11%(0.3 mg/kg), 0(1 mg/kg), 0(3 mg/kg), 0(10 mg/kg), 4% (all patients); Dose-expansion: Grade 3–4 trAE:19%, serious trAEs: 4%
**PD-1/PD-L1 inhibitor plus anti-VEGF**								
IMbrave150 (Finn et al., 2020; Cheng et al., 2022) [12, 107] NCT03434379	Phase 3 1st	N = 501 (336 vs. 165)	BCLC Stage A-C; ECOG PS of 0 or 1; Child-Pugh score of A.	Atezolizumab plus bevacizumab vs. Sorafenib		mOS: 19.2 vs. 13.4 months, HR = 0.66 95% CI 0.52–0.85, descriptive p < 0.001; mPFS: 6.9 vs. 4.3 months, HR = 0.65 95% CI 0.53–0.81, descriptive p < 0.001; ORR: 30% vs. 11%		Grade 3–4 trAE: 43% vs. 46%; serious trAE: 23% vs. 16%
ORIENT-32 (Ren et al., 2021) [110] NCT03794440	Phase 2-3 1st	N = 595 (Phase 2: 24; Phase 3: 380 vs. 191)	BCLC Stage B/C; ECOG PS of 0 or 1; Child-Pugh score of B7 or less.	Sintilimab plus IBI305 vs. Sorafenib		Phase 3 part: mPFS: 4.6 months vs. 2.8 months HR = 0.56; 95% CI 0.46–0.70; p < 0.001. mOS: not reached vs. 10.4 months, HR = 0.57; 95% CI 0.43–0.75; p < 0.001. ORR: 21% vs. 4%.		Phase 2 part: grade 3–4 trAEs:29%; serious trAEs: 25% Phase 3 part: grade 3 trAEs:34% vs. 36%; Serious trAEs:17% vs. 10%.
GO30140 (Lee et al., 2020) [15] NCT02715531	Phase 1b 1st	N = 223 (Group A: 104; Group F: 60 vs. 59)	ECOG PS of 0 or 1; BCLC A4, B, C; group A: Child-Pugh score up to B7; group F: Child-Pugh score of A.	Group A: Atezolizumab plus bevacizumab vs. Bevacizumab Group F: Atezolizumab plus bevacizumab vs. Atezolizumab		Group A: ORR: 36%; Group F: mPFS: 5.6 vs. 3.4 months; HR = 0.55, 80% CI 0.40–0.74, p = 0.011; ORR: 20% vs. 17%	Group A: serious trAEs:24%; group F: serious trAEs: 25% vs. 10%	
**PD-1/PD-L1 inhibitor plus TKI**								
CARES-310 (Qin et al., 2023) [112] NCT03764293	Phase 3 1st	N = 543 (272 vs. 271)	BCLC Stage B/C; ECOG PS of 0 or 1; Child-Pugh score of A.	Camrelizumab plus rivoceranib vs. Sorafenib		mPFS: 5.6 months vs. 3.7 months; mOS: 22.1 months vs. 15.2 months; ORR: 25% months vs. 6%	Grade ≥ 3 trAEs: 80.9% vs. 52.4%	
LEAP-002 (Finn et al., 2022) [113] NCT03713593	Phase 3 1st	N = 794 (395 vs. 399)	BCLC Stage B/C; ECOG PS of 0 or 1; Child-Pugh score of A.	Lenvatinib plus pembrolizumab vs. Lenvatinib plus placebo		mOS: 21.2 vs. 19.0 months, HR = 0.840, 95% CI 0.708–0.997, p = 0.0227 mPFS: 8.2 vs. 8.0 months, HR 0.867, 95% CI 0.734–1.024, p = 0.0466 ORR: 26.1% vs. 17.5%	Grade 3–5 trAE: 62.5% vs. 57.5%	
COSMIC-312 (Kelly et al., 2022) [114] NCT03755791	Phase 3 1st	N = 837 (432 vs. 217 vs. 188)	BCLC Stage B/C; ECOG PS of 0 or 1; Child-Pugh score of A.	Cabozantinib plus atezolizumab vs. Sorafenib vs. Cabozantinib		mPFS at final analysis: 6.8 months (Cabozantinib + Atezolizumab), 4.2 months (Sorafenib), HR = 0.63; 99% CI 0.44–0.91, p = 0.0012 mOS at interim analysis: 15.4 months (Cabozantinib + Atezolizumab),15.5 months (Sorafenib), HR = 0.90; 96% CI 0.69–1.18, p = 0.44 mPFS at interim analysis: 5.8 months (Cabozantinib), 4.3 months (Sorafenib), HR = 0.71, 99% CI 0.51–1.01, p = 0.011	Grade 3 trAE: 51% vs. 30% vs. 52%; grade 4 trAE: 3% vs. 2% vs. 3%; serious trAE: 18% vs. 8% vs. 13%.	
RESCUE (Xu et al., 2021) [111] NCT03463876	Phase 2 2nd	N = 120 (First line: 70; second line: 120)	BCLC Stage B/C; ECOG PS of 0 or 1; Child-Pugh score of A.	Camrelizumab plus apatinib		ORR: 34.3% (first line), 22.5% (second line); mPFS: 5.7 months (first line), 5.5 months (second line).	Grade 3–5 trAE: 78.6% (first line), 76.7% (second line), 77.4%(total); Serious trAE: 32.9% (first line), 26.7% (second line), 28.9% (total)	
TIS plus LEN (Xu et al., 2022) [115] NCT04401800	Phase 2 1st	N = 64	BCLC Stage B/C; ECOG PS of 0 or 1; Child-Pugh score of A.	Tislelizumab plus lenvatinib		ORR: 38.7%; mPFS: 9.6 months	Grade ≥ 3 trAEs: 28.1%, serious trAE: 9.4%	
KEYNOTE-524 (Zhu et al., 2020) NCT03006926	Phase 1b 1st	N = 100	BCLC Stage B/C; ECOG PS of 0 or 1; Child-Pugh score of A.	Lenvatinib plus pembrolizumab		ORR: 36%; mPFS:8.6 months; mOS: 22.0 months	Grade ≥ 3 trAEs: 67%, serious trAE: 36%	
**PD-1/PD-L1 inhibitor plus CTLA-4 inhibitor**								
HIMALAYA (Abou-Alfa et al., 2022) [19] NCT03298451	Phase 3 1st	N = 1171 (393 vs. 389 vs.389)	BCLC Stage B/C; ECOG PS of 0 or 1; Child-Pugh score of A.	Single Tremelimumab Regular Interval Durvalumab (STRIDE) vs. Durvalumab vs. Sorafenib		mOS: 16.43 vs. 16.56 vs. 13.77 months (STRIDE vs. Sorafenib: HR = 0.78, 96.02% CI, 0.65–0.93; Durvalumab vs. Sorafenib: HR = 0.86, 95.67% CI, 0.73–1.03) mPFS: 3.78 vs. 3.65 vs. 4.07 months (STRIDE vs. Sorafenib: HR = 0.78, 95% CI, 0.65–0.93; Durvalumab vs. Sorafenib: HR = 0.86, 95% CI, 0.73–1.03); ORR: 20.1% vs. 17.0% vs. 5.1%	Grade 3–4 trAEs: 25.8% vs. 12.9% vs. 36.9%; Serious trAEs: 17.5% vs. 8.2% vs. 9.4%	
CheckMate 040 (Yau et al., 2020) [17] NCT01658878	Phase 1/2 2nd	N = 148 (Arm A: 50; Arm B: 49; Arm C: 49)	BCLC Stage A/B/C; ECOG PS of 0 or 1; Child-Pugh score of A.	Arm A: Nivolumab 1 mg/kg plus ipilimumab 3 mg/kg Q3W (4 doses) followed by nivolumab 240 mg intravenously Q2W; Arm B: Nivolumab 3 mg/kg plus ipilimumab 1 mg/kg Q3W (4 doses) followed by nivolumab 240 mg intravenously Q2W; Arm C: Nivolumab 3 mg/kg Q2W plus ipilimumab 1 mg/kg Q6W.		ORR: 32% (Arm A), 27% (Arm B), 29% (Arm C); mOS: 22.8 months (Arm A), 12.5 months (Arm B), 12.7 months (Arm C)	Grade 3–4 trAE: 53% (Arm A), 29% (Arm B), 31% (Arm C)	
**PD-L1 inhibitor plus anti-VEGF and anti-TIGIT**								
MORPHEUS-liver (Finn et al., 2023) [108] NCT04524871	Phase 1b/2 1st	N = 58 (40 vs. 18)	BCLC Stage B/C; ECOG PS of 0 or 1; Child-Pugh score of A.	Tiragolumab + Atezolizumab + Bevacizumab vs. Atezolizumab + Bevacizumab		ORR: 42.5% vs. 11.1%; mPFS: 11.1 vs. 4.2 months	Grade 3–4 trAEs: 27.5% vs. 33.3%	

Abbreviations: AFP, alpha-fetoprotein; AE, adverse event; aHCC, advanced hepatocellular carcinoma; BCLC, Barcelona clinic liver cancer; CI, confidence interval; CTLA-4, cytotoxic T lymphocyte–associated antigen 4; DCR, disease control rate; ECOG PS, ECOG Performance Status; HBV, hepatitis B virus; HCV, hepatitis C virus; HR, hazard ratio; mOS, median overall survival; mPFS, median progression-free survival; ORR, objective response rate; PD-1, programmed death-1; PD-L1, programmed death-1; STRIDE, Single Tremelimumab Regular Interval Durvalumab; TIGIT, T cell immunoglobulin and ITIM domain; TKI, tyrosine kinase inhibitor; teAE, treatment-emergent adverse event; trAE, treatment-related adverse event; VEGF, vascular endothelial growth factor

The recently developed bispecific antibody (BsAb) drug (i.e., AK104) has shown potential in solid tumors [116]. Consequently, research on such agents is being conducted in aHCC. Additionally, novel combinations of immunotherapeutic agents are being explored for the treatment of aHCC, with a focus on newly developed ICIs such as TIGIT and LAG3 inhibitors. The umbrella study (NCT04524871) serves as a representative example of these endeavors. Furthermore, several prospective clinical studies focus on the combinations of local therapies (e.g., transarterial chemoembolization) with immunotherapy in intermediate HCC. Noteworthy studies in this area include LEAP-012, ABC-HCC, and EMERALD. The effectiveness of this regimen has already been demonstrated in the retrospective study [39]. Moreover, prospective research on the combination of stereotactic radiation therapy with immunotherapy is currently unfolding. The key ongoing clinical trials studying immunotherapies for unresectable HCC are listed in Table 4.

**Table 4:** Key ongoing advanced hepatocellular carcinoma immunotherapy clinical trials

NCT number	Phase (Estimated Number); Line	Target population (Region)	Regimen	Primary endpoint	Status (Estimated Completion Date)	Categories	Therapeutic reagent types
NCT04194775	III (N = 534); 1st	Histologically, cytologically, or clinically confirmed; BCLC stage B/C (Global)	CS1003(PD-1 inhibitor) + Lenvatinib vs. Placebo + Lenvatinib	OS	Active, not recruiting (June 30, 2025)	Anti-PD-1 + TKI	IgG4 mAb + TKI
NCT04523493	III (N = 530); 1st	Histological or cytologically confirmed; BCLC stage B/C (Global)	Toripalimab (PD-1 inhibitor) + Lenvatinib vs. Placebo + Lenvatinib	OS	Active, not recruiting (September 1, 2026)	Anti-PD-1 + TKI	IgG4 mAb + TKI
CTR20200192	III (N = 528); 1st	Histological or cytologically confirmed; BCLC stage B/C (Global)	JS001 (PD-1 inhibitor) + Bevacizumab vs. Sorafenib	OS; PFS	Recruiting (NA)	Anti-PD-1 + anti-VEGF	IgG4 mAb + IgG1 mAb
NCT04560894	II/III (N = 621); 1st	Unresectable HCC; BCLC stage B/C (China)	SCT-I10A (PD-1 inhibitor) + SCT510 vs. Sorafenib	OS; PFS	Recruiting (September 2024)	Anti-PD-1 + anti-VEGF	IgG4 mAb + mAb
NCT05603039	Ib/II (N = 80); 1st	Histologically or clinically confirmed (China)	QL1604 (PD-1 inhibitor) + Bevacizumab	ORR	Recruiting (December 30, 2023)	Anti-PD-1 + anti-VEGF	IgG4 mAb + IgG1 mAb
NCT04444167	Ib/II (N = 30); 1st	Histological or cytologically confirmed; BCLC stage B/C (China)	AK104 (PD-1/CTLA-4 bispecific antibody) + Lenvatinib	ORR	Recruiting (October 30, 2023)	PD-1/CTLA-4 bispecific antibody + TKI	IgG1 tetrameric BsAb + TKI
NCT05603039	Ib/II (N = 80); 1st	Histologically or clinically confirmed (China)	QL1706 (PD-1/CTLA-4 two engineered monoclonal antibodies) + Bevacizumab	ORR	Recruiting (December 30, 2023)	PD-1/CTLA-4 bispecific antibody + anti-VEGF	Single bifunctional MabPair product + IgG1 mAb
NCT04542837	II (N = 55); 1st	Histological or cytologically confirmed; BCLC stage B/C (China)	KN046 (PD-L1 /CTLA-4 bispecific antibody) + Lenvatinib	ORR	Recruiting (May 21, 2023)	PD-L1/CTLA-4 bispecific antibody + TKI	IgG1 Fc BsAb + TKI
NCT04948697	II (N = 90); 1st	Histologically confirmed; BCLC stage B/C (China)	Ociperlimab (anti-TIGIT) + Tislelizumab + BAT1706(anti-VEGF) vs. Tislelizumab + BAT1706	ORR	Active, not recruiting (August 2023)	Anti-TIGIT + anti-PD-1 + anti-VEGF	IgG1 mAb + IgG4 mAb + mAb
NCT04524871 (Morpheus-Liver)	Ib/II (N = 400); 1st	Histologically, cytologically, or clinically confirmed; advanced HCC (Global)	Atezolizumab (PD-L1 inhibitor) + Bevacizumab + Tiragolumab (anti-TIGIT antibody) Atezolizumab (PD-L1 inhibitor) + Bevacizumab + Tocilizumab Atezolizumab (PD-L1 inhibitor) + Bevacizumab + TPST-1120 (PPARα antagonist) Bevacizumab + RO7247669 (PD1-LAG3 Bispecific Antibody) Atezolizumab (PD-L1 inhibitor) + Bevacizumab + ADG126 (anti-CTLA-4 SAFEbody) Atezolizumab + Bevacizumab	ORR	Recruiting (December 27, 2025)	Umbrella study	Atezolizumab: IgG1 mAb; Bevacizumab: IgG1 mAb; Tiragolumab: IgG1/kappa mAb; TPST-1120: oral PPARα antagonist; RO7247669: BsAb; ADG126: masked anti-CTLA-4 SAFEbody
NCT05904886 (IMbrave152)	III (N = 650); 1st	Histologically or cytologically confirmed; locally advanced or metastatic and/or unresectable HCC (United States)	Tiragolumab (anti-TIGIT antibody) + Atezolizumab (PD-L1 inhibitor) + Bevacizumab vs. Atezolizumab + Bevacizumab + Placebo	OS, PFS	Not yet recruiting (September 1, 2026)	Anti-TIGIT + anti-PD-L1 + anti-VEGF	IgG1 kappa mAb + IgG1 mAb + IgG1 mAb
NCT04246177 (LEAP-012)	III (N = 450); 1st	Radiology, histology, or cytology confirmed; intermediate stage (Global)	Pembrolizumab (PD-1 inhibitor) + Lenvatinib + TACE vs. Placebo + TACE	OS, PFS	Active, not recruiting (December 31, 2029)	Anti-PD-1 + TKI + TACE	TKI + IgG4 kappa mAb + locoregional therapy
NCT04777851 (REPLACE)	III (N = 496); 1st	Radiology, histology, or cytology confirmed; intermediate stage (Global)	Nivolumab (PD-1 inhibitor) + Regorafenib vs. TACE	PFS	Not yet recruiting (April 2027)	Anti-PD-1 + TKI vs. TACE	IgG4 mAb + TKI
NCT04803994 (ABC-HCC)	III (N = 434); 1st	Histological or radiology confirmed; intermediate stage (Global)	Atezolizumab (PD-L1 inhibitor) + Bevacizumab vs. TACE	Time to failure of treatment strategy	Recruiting (April 1, 2025)	Anti-PD-L1 + anti-VEGF vs. TACE	IgG1 mAb + IgG1 mAb + locoregional therapy
			Atezolizumab (PD-L1 inhibitor) + Bevacizumab + TACE				
NCT04712643 (ML-42,612)	III (N = 342); 1st	Histology/ cytology confirmed (Global)	Atezolizumab (PD-L1 inhibitor) + Bevacizumab + TACE vs. TACE	PFS; OS	Recruiting (February 28, 2029)	Anti-PD-L1 + anti-VEGF + TACE	IgG1 mAb + IgG1 mAb + locoregional therapy
NCT03778957 (EMERALD-1)	III (N = 724); 1st	Intermediate stage; without extrahepatic disease or main portal vein thrombosis (Vp3/Vp4) (Global)	Arm A: TACE + Durvalumab (PD-L1 inhibitor)	PFS for Arm B vs. Arm C	Active, not recruiting (August 19, 2024)	Anti-PD-L1 + anti-VEGF + TACE	IgG1 kappa mAb + IgG1 mAb + locoregional therapy
			Arm B: TACE + Durvalumab (PD-L1 inhibitor) + Bevacizumab				
			Arm C: TACE + Placebo				
NCT04224636 (DEMAND)	II (N = 106); 1st	Histologically confirmed (Germany)	Up-front Atezolizumab (PD-L1 inhibitor) + Bevacizumab, then TACE	24-months survival rate	Recruiting (March 1, 2025)	Anti-PD-L1 + anti-VEGF + TACE	IgG1 mAb + IgG1 mAb + locoregional therapy
NCT05301842 (EMERALD-3)	III (N = 525); 1st	Locoregional HCC (Global)	Arm A: TACE + Durvalumab (PD-L1 inhibitor) + Tremelimumab + Lenvatinib	PFS for Arm A vs. Arm C	Recruiting (February 26, 2027)	Anti-PD-L1 + anti-CTLA-4 + TKI + TACE	IgG1 kappa mAb + IgG2 mAb + TKI + locoregional therapy
			Arm B: TACE + Durvalumab (PD-L1 inhibitor) + Tremelimumab				
			Arm C: TACE				
NCT05063565 (ROWAN)	II (N = 100); 1st	Radiology or cytology confirmed; portal vein thrombosis (Vp0, Vp1, or Vp2); intermediate stage (United States, Spain)	SIRT followed STRIDE	ORR	Recruiting (June 2027)	Anti-PD-L1 + anti-CTLA-4 + SIRT	IgG1 kappa mAb + IgG2 mAb + radiation therapy
NCT04770896 (IMbrave251)	III (N = 554); 2nd	Histologically, cytologically, or clinically confirmed; advanced HCC (Global)	Atezolizumab (PD-L1 inhibitor) + Lenvatinib/ Sorafenib vs. Lenvatinib/ Sorafenib alone	OS	Recruiting (September 23, 2024)	Anti-PD-L1 + TKI	IgG1 mAb + TKI
NCT04696055	II (N = 95); 2nd	Histological or cytological confirmed; BCLC stage B/ C (Global)	Pembrolizumab (PD-1 inhibitor) + Regorafenib	ORR	Active, not recruiting (May 15, 2024)	Anti-PD-1 + TKI	IgG4 kappa mAb + TKI
NCT05873244	II(N = 44); 2nd and more	Prior treatment with systemic treatment consisting of immune checkpoint inhibitors; Child-Pugh class A; ECOG PS of 0 or 1.	Geptanolimab (PD-1 inhibitor) + Zabadinostat (class 1 HDAC inhibitor) vs. Lenvatinib/ Sorafenib alone	PFS	Recruiting (December 30, 2027)	Anti-PD-1 + HDAC inhibitor	IgG4 mAb + HDAC inhibitor

Abbreviations: BsAb, bispecific antibody; CTLA-4, Cytotoxic T lymphocyte-associated antigen 4; HCC, hepatocellular carcinoma; HDAC, histone deacetylase; IgG, Immunoglobulin G; mAb, monoclonal antibody; ORR, objective response rate; OS, overall survival; PD-1, programmed death-1; PD-L1, programmed death ligand 1; PFS, progression-free survival; PPARα, peroxisome proliferator-activated receptor alpha; SIRT, selective internal radiation therapy; STRIDE, Single Tremelimumab Regular Interval Durvalumab; TACE, transarterial chemoembolization; TIGIT, T cell immunoglobulin and ITIM domain; TKI, tyrosine kinase inhibitor; trAE, treatment-related adverse event; VEGF, anti-vascular endothelial growth factor

## Conclusions and perspectives

**Fig. 1:**
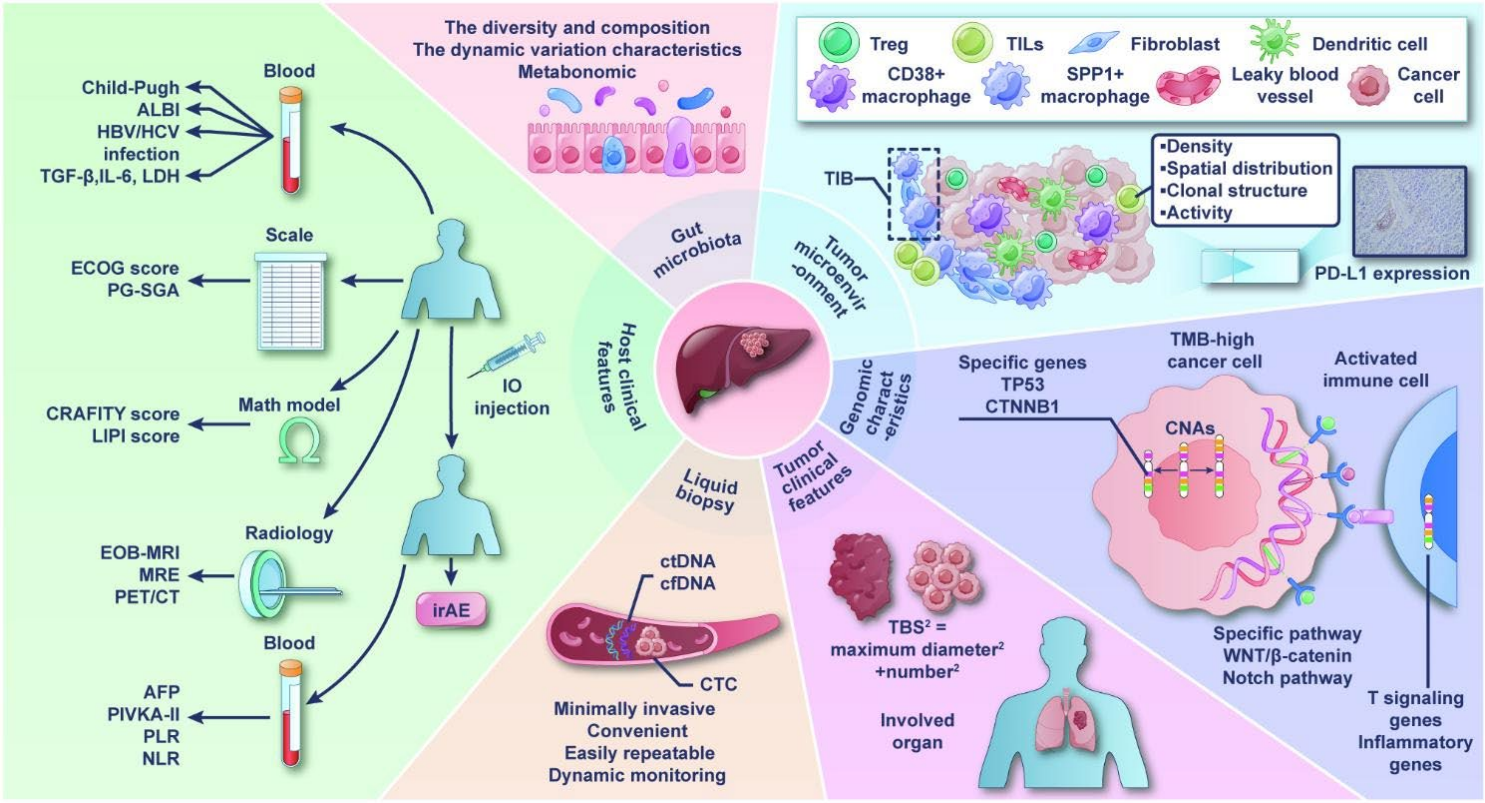
The summary of the biomarkers in PD-1/PD-L1 inhibitor-based therapy in aHCC. Current studies on biomarkers are focused on the tumor microenvironment, tumor genomics, tumor clinical features, host clinical features, liquid biopsy, and gut microbiota. Abbreviations: AFP, alpha-fetoprotein; aHCC, advanced hepatocellular carcinoma; ALBI, albumin-bilirubin; cfDNA, cell-free DNA; CNAs, copy number alterations; CTC, circulating tumor cell; ctDNA, circulating tumor DNA; ECOG, Eastern Cooperative Oncology Group; EOB-MRI, Gd-EOB-DTPA-enhanced magnetic resonance imaging; HBV, hepatitis B virus; HCV, hepatitis C virus; IL-6, interleukin-6; IO, immunotherapy; irAE, immune-related adverse event; LDH, lactate dehydrogenase; MRE, magnetic resonance elastography; NLR, neutrophil-lymphocyte ratio; PD-1, programmed death-1; PD-L1, programmed death ligand 1; PET/CT, positron emission tomography-computed tomography; PG-SGA, patient-generated subjective global assessment; PIVKA-II, abnormal prothrombin; PLR, platelet-to-lymphocyte ratio; TBS, tumor burden score; TGF-β, Transforming Growth Factor beta; TIB, tumor immune barrier; TILs, tumor-infiltrating lymphocytes; Treg, regulatory T cell; TMB, tumor mutational burden

Systemic therapy using PD-1/PD-L1 inhibitors has been shown to be effective in treating HCC; however, this treatment is only beneficial to a subset of patients. Therefore, biomarker analysis is crucial for identifying individuals who will most likely respond to this treatment. A summary of the aforementioned biomarkers is shown in Fig. 1 and Supplementary Table 1.

Despite the importance of biomarkers in HCC, their use faces several challenges. First, the methods used for immunotherapy lack uniformity. As more studies combine PD-1/PD-L1 inhibitors with TKI/VEGF therapy, the underlying mechanisms and effectiveness may vary. Second, although some cases of HCC can be diagnosed through imaging, pathological tissue may not be available in all cases, thus increasing the difficulty of analyzing the immune microenvironment. Limited biomarkers are available for dynamic monitoring, and data are scarce for adjusting treatment after drug resistance.

With continued advances in research on HCC immunotherapy, mainly through extensive sample studies and subsequent subgroup analyses, biomarkers will hopefully become more widespread, which will allow for earlier identification of the target population. In the future, cutting-edge non-invasive monitoring methods (such as ctDNA), imaging parameters (such as PET/CT), and multi-dimensional information from artificial intelligence radiomics and single-cell sequencing sources may help us to comprehensively understand the mechanisms behind HCC immunotherapy response and the causes of drug resistance. These findings will ultimately result in more tailored treatment options.

### Electronic supplementary material

Below is the link to the electronic supplementary material.

Supplementary Material 1

## Data Availability

The material supporting the information in this review has been included in this article.

## List of Abbreviations

^18^F-FDG: ^18^F-fluorodeoxyglucose
^68^Ga-FAPI: ^68^Ga-labeled FAP inhibitor
AFP: Alpha-fetoprotein
aHCC: Advanced hepatocellular carcinoma
ALBI: Albumin-bilirubin
Atezo/Bev: Atezolizumab and bevacizumab
BsAb: Bispecific antibody
CCF: ctDNA content fraction
cDC1: conventional DC 1
cfDNA: cell-free DNA
CNA: Copy number alteration
CNV: Copy number variation
CPS: Combined positive score
CR: Complete response
CRP: C-reactive protein
CTCs: Circulating tumor cells
ctDNA: circulating tumor DNA
CTLA-4: Cytotoxic T lymphocyte–associated antigen 4
CYT: Cytolytic Activity Score
CAFs: Cancer-associated fibroblasts
dNLR: derived neutrophil-lymphocyte ratio
ECOG: Eastern Cooperative Oncology Group
Gd-EOB-DTPA: Gadolinium ethoxybenzyl diethylenetriamine pentaacetic acid
EOB-MRI: Gd-EOB-DTPA-enhanced magnetic resonance imaging
HCC: Hepatocellular carcinoma
HPD: Hyperprogressive disease
ICI: Immune checkpoint inhibitor
IFN-γ: Interferon γ
IL-6: Interleukin-6
irAEs: immune-related adverse events
LDH: Lactate dehydrogenase
MRE: Magnetic resonance elastography
nsSNVs: nonsense single nucleotide mutations
ORR: Objective response rate
OS: Overall survival
PD-1: Programmed death-1
PD-L1: Programmed death ligand 1
PET/CT: Positron emission tomography-computed tomography
PFS: Progression-free survival
PG-SGA: Patient-generated subjective global assessment
PIVKA-II: Prothrombin induced by vitamin K absence-II
PLR: Platelet-to-lymphocyte ratio
PR: Partial response
RCTs: Randomized clinical trials
RLTCC: Ratio of lymphocyte to total cell count
SD: Stable disease
SDC: Stimulatory dendritic cell
STRIDE: Single Tremelimumab Regular Interval Durvalumab
TBS: Tumor burden score
TCGA-LIHC: The Cancer Genome Atlas Program Liver Hepatocellular Carcinoma
TILs: Tumor-infiltrating lymphocytes
TIB: Tumor immune barrier
TIGIT: T cell immunoglobulin and ITIM domain
TKIs: Tyrosine kinase inhibitors
TMB: Tumor mutational burden
TME: Tumor microenvironment
Treg: Regulatory T cell
TPS: Tumor proportion score
VEGF: Vascular endothelial growth factor

## References

[1] European Association for the Study of the Liver (2018). Electronic address eee, European Association for the study of the L. EASL Clinical Practice guidelines: management of hepatocellular carcinoma. J Hepatol.

[2] Villanueva A, Hepatocellular Carcinoma (2019). N Engl J Med.

[3] Zhou J, Sun HC, Wang Z, Cong WM, Wang JH, Zeng MS (2018). Guidelines for diagnosis and treatment of primary Liver Cancer in China (2017 Edition). Liver Cancer.

[4] Ringelhan M, Pfister D, O’Connor T, Pikarsky E, Heikenwalder M (2018). The immunology of hepatocellular carcinoma. Nat Immunol.

[5] El-Khoueiry AB, Sangro B, Yau T, Crocenzi TS, Kudo M, Hsu C (2017). Nivolumab in patients with advanced hepatocellular carcinoma (CheckMate 040): an open-label, non-comparative, phase 1/2 dose escalation and expansion trial. Lancet.

[6] Zhu AX, Finn RS, Edeline J, Cattan S, Ogasawara S, Palmer D (2018). Pembrolizumab in patients with advanced hepatocellular carcinoma previously treated with sorafenib (KEYNOTE-224): a non-randomised, open-label phase 2 trial. Lancet Oncol.

[7] Sangro B, Park J, Finn R, Cheng A, Mathurin P, Edeline J (2020). LBA-3 CheckMate 459: long-term (minimum follow-up 33.6 months) survival outcomes with nivolumab versus sorafenib as first-line treatment in patients with advanced hepatocellular carcinoma. Ann Oncol.

[8] Finn RS, Ryoo BY, Merle P, Kudo M, Bouattour M, Lim HY, et al. Pembrolizumab as Second-Line therapy in patients with Advanced Hepatocellular Carcinoma in KEYNOTE-240: a Randomized, Double-Blind, phase III trial. J Clin Oncol. 2019. Jco1901307. 10.1200/JCO.19.01307 31790344

[9] Chen Y, Ramjiawan RR, Reiberger T, Ng MR, Hato T, Huang Y (2015). CXCR4 inhibition in Tumor microenvironment facilitates anti-programmed death receptor-1 immunotherapy in sorafenib-treated hepatocellular carcinoma in mice. Hepatology.

[10] Shigeta K, Datta M, Hato T, Kitahara S, Chen IX, Matsui A et al. Dual PD-1 and VEGFR-2 blockade promotes vascular normalization and enhances anti-tumor immune responses in HCC. Hepatology. 2019. 10.1002/hep.30889 PMC7000304 31378984

[11] Shigeta K, Matsui A, Kikuchi H, Klein S, Mamessier E, Chen IX et al. Regorafenib combined with PD1 blockade increases CD8 T-cell infiltration by inducing CXCL10 expression in hepatocellular carcinoma. J Immunother Cancer. 2020;8(2). 10.1136/jitc-2020-001435 PMC7689089 33234602

[12] Finn RS, Qin S, Ikeda M, Galle PR, Ducreux M, Kim TY (2020). Atezolizumab plus Bevacizumab in Unresectable Hepatocellular Carcinoma. N Engl J Med.

[13] Havel JJ, Chowell D, Chan TA (2019). The evolving landscape of biomarkers for checkpoint inhibitor immunotherapy. Nat Rev Cancer.

[14] Yau T, Park JW, Finn RS, Cheng AL, Mathurin P, Edeline J (2022). Nivolumab versus Sorafenib in advanced hepatocellular carcinoma (CheckMate 459): a randomised, multicentre, open-label, phase 3 trial. Lancet Oncol.

[15] Lee MS, Ryoo BY, Hsu CH, Numata K, Stein S, Verret W (2020). Atezolizumab with or without bevacizumab in unresectable hepatocellular carcinoma (GO30140): an open-label, multicentre, phase 1b study. Lancet Oncol.

[16] Qin S, Ren Z, Meng Z, Chen Z, Chai X, Xiong J (2020). Camrelizumab in patients with previously treated advanced hepatocellular carcinoma: a multicentre, open-label, parallel-group, randomised, phase 2 trial. Lancet Oncol.

[17] Yau T, Kang YK, Kim TY, El-Khoueiry AB, Santoro A, Sangro B et al. Efficacy and safety of Nivolumab Plus Ipilimumab in patients with Advanced Hepatocellular Carcinoma previously treated with Sorafenib: the CheckMate 040 Randomized Clinical Trial. JAMA Oncol. 2020. 10.1001/jamaoncol.2020.4564 PMC7530824 33001135

[18] Lee DW, Cho EJ, Lee JH, Yu SJ, Kim YJ, Yoon JH et al. Phase II study of Avelumab in patients with Advanced Hepatocellular Carcinoma Previously Treated with Sorafenib. Clin Cancer Res. 2020. 10.1158/1078-0432.CCR-20-3094 33139266

[19] Abou-Alfa GK, Lau G, Kudo M, Chan SL, Kelley RK, Furuse J (2022). Tremelimumab plus Durvalumab in Unresectable Hepatocellular Carcinoma. NEJM Evid.

[20] Zhu J, Armstrong AJ, Friedlander TW, Kim W, Pal SK, George DJ (2018). Biomarkers of immunotherapy in urothelial and renal cell carcinoma: PD-L1, Tumor mutational burden, and beyond. J Immunother Cancer.

[21] Riaz N, Havel JJ, Makarov V, Desrichard A, Urba WJ, Sims JS (2017). Tumor and Microenvironment Evolution during Immunotherapy with Nivolumab. Cell.

[22] Sangro B, Melero I, Wadhawan S, Finn RS, Abou-Alfa GK, Cheng AL (2020). Association of inflammatory biomarkers with clinical outcomes in nivolumab-treated patients with advanced hepatocellular carcinoma. J Hepatol.

[23] Duffy AG, Ulahannan SV, Makorova-Rusher O, Rahma O, Wedemeyer H, Pratt D (2017). Tremelimumab in combination with ablation in patients with advanced hepatocellular carcinoma. J Hepatol.

[24] Ng HHM, Lee RY, Goh S, Tay ISY, Lim X, Lee B et al. Immunohistochemical scoring of CD38 in the Tumor microenvironment predicts responsiveness to anti-PD-1/PD-L1 immunotherapy in hepatocellular carcinoma. J Immunother Cancer. 2020;8(2). 10.1136/jitc-2020-000987 PMC7451957 32847986

[25] Lee H-S, Kang KKG, Jung K-H, Kaseb AO, Lee SS. Quantitative analysis of spatial distribution of lymphocytes in hepatocellular carcinoma: a biomarker correlated with survival and gene expression in cancer immune system. J Clin Oncol. 2022;40(abstr 4119).

[26] Agdashian D, ElGindi M, Xie C, Sandhu M, Pratt D, Kleiner DE (2019). The effect of anti-CTLA4 treatment on peripheral and intra-tumoral T cells in patients with hepatocellular carcinoma. Cancer Immunol Immunother.

[27] Balli D, Rech AJ, Stanger BZ, Vonderheide RH (2017). Immune Cytolytic Activity Stratifies Molecular subsets of Human Pancreatic Cancer. Clin Cancer Res.

[28] Narayanan S, Kawaguchi T, Yan L, Peng X, Qi Q, Takabe K (2018). Cytolytic activity score to assess Anticancer Immunity in Colorectal Cancer. Ann Surg Oncol.

[29] Takahashi H, Kawaguchi T, Yan L, Peng X, Qi Q, Morris LGT et al. Immune Cytolytic Activity for Comprehensive understanding of Immune Landscape in Hepatocellular Carcinoma. Cancers (Basel). 2020;12(5). 10.3390/cancers12051221 PMC7281225 32414098

[30] Salmon H, Idoyaga J, Rahman A, Leboeuf M, Remark R, Jordan S (2016). Expansion and activation of CD103(+) dendritic cell progenitors at the Tumor Site enhances Tumor responses to therapeutic PD-L1 and BRAF inhibition. Immunity.

[31] Barry KC, Hsu J, Broz ML, Cueto FJ, Binnewies M, Combes AJ (2018). A natural killer-dendritic cell axis defines checkpoint therapy-responsive Tumor microenvironments. Nat Med.

[32] Zhu AX, Guan Y, Abbas AR, Koeppen H, Lu S, Hsu C-H (2020). Abstract CT044: genomic correlates of clinical benefits from atezolizumab combined with bevacizumab vs. atezolizumab alone in patients with advanced hepatocellular carcinoma (HCC). Cancer Res.

[33] Cui X, Han L, Cui L, Fu G, Liu E, Wang D (2023). Immune index: a gene and cell prognostic signature for immunotherapy response prediction in hepatocellular carcinoma. Pharmacol Res.

[34] Ma L, Hernandez MO, Zhao Y, Mehta M, Tran B, Kelly M (2019). Tumor Cell Biodiversity drives Microenvironmental Reprogramming in Liver Cancer. Cancer Cell.

[35] Ma L, Wang L, Khatib SA, Chang CW, Heinrich S, Dominguez DA (2021). Single-cell atlas of Tumor cell evolution in response to therapy in hepatocellular carcinoma and intrahepatic cholangiocarcinoma. J Hepatol.

[36] Xue R, Zhang Q, Cao Q, Kong R, Xiang X, Liu H (2022). Liver tumour immune microenvironment subtypes and neutrophil heterogeneity. Nature.

[37] Liu Y, Xun Z, Ma K, Liang S, Li X, Zhou S (2023). Identification of a tumour immune barrier in the HCC microenvironment that determines the efficacy of immunotherapy. J Hepatol.

[38] Zhang S, Yuan L, Danilova L, Mo G, Zhu Q, Deshpande A (2023). Spatial transcriptomics analysis of neoadjuvant cabozantinib and nivolumab in advanced hepatocellular carcinoma identifies Independent mechanisms of resistance and recurrence. Genome Med.

[39] Zhu HD, Li HL, Huang MS, Yang WZ, Yin GW, Zhong BY (2023). Transarterial chemoembolization with PD-(L)1 inhibitors plus molecular targeted therapies for hepatocellular carcinoma (CHANCE001). Signal Transduct Target Ther.

[40] Llovet JM, Vogel A, Madoff DC, Finn RS, Ogasawara S, Ren Z (2022). Randomized Phase 3 LEAP-012 study: Transarterial Chemoembolization with or without Lenvatinib Plus Pembrolizumab for Intermediate-Stage Hepatocellular Carcinoma not amenable to curative treatment. Cardiovasc Intervent Radiol.

[41] Ben Khaled N, Seidensticker M, Ricke J, Mayerle J, Oehrle B, Rössler D (2022). Atezolizumab and bevacizumab with transarterial chemoembolization in hepatocellular carcinoma: the DEMAND trial protocol. Future Oncol.

[42] Li L, Rao X, Wen Z, Ding X, Wang X, Xu W (2020). Implications of driver genes associated with a high Tumor mutation burden identified using next-generation sequencing on immunotherapy in hepatocellular carcinoma. Oncol Lett.

[43] Llovet JM, Montal R, Sia D, Finn RS (2018). Molecular therapies and precision medicine for hepatocellular carcinoma. Nat Rev Clin Oncol.

[44] Bassaganyas L, Pinyol R, Esteban-Fabró R, Torrens L, Torrecilla S, Willoughby CE (2020). Copy-number Alteration Burden differentially impacts Immune profiles and Molecular features of Hepatocellular Carcinoma. Clin Cancer Res.

[45] Long J, Wang A, Bai Y, Lin J, Yang X, Wang D (2019). Development and validation of a TP53-associated immune prognostic model for hepatocellular carcinoma. EBioMedicine.

[46] Ruiz de Galarreta M, Bresnahan E, Molina-Sanchez P, Lindblad KE, Maier B, Sia D (2019). beta-catenin activation promotes Immune Escape and resistance to Anti-PD-1 therapy in Hepatocellular Carcinoma. Cancer Discov.

[47] Harding JJ, Nandakumar S, Armenia J, Khalil DN, Albano M, Ly M et al. Prospective genotyping of Hepatocellular Carcinoma: clinical implications of Next Generation sequencing for matching patients to targeted and Immune therapies. Clin Cancer Res. 2018. 10.1158/1078-0432.CCR-18-2293 PMC6689131 30373752

[48] von Felden J, Craig AJ, Garcia-Lezana T, Labgaa I, Haber PK, D’Avola D et al. Mutations in circulating Tumor DNA predict primary resistance to systemic therapies in advanced hepatocellular carcinoma. Oncogene. 2020. 10.1038/s41388-020-01519-1 PMC12452111 33097857

[49] Zhu AX, Guan Y, Abbas AR, Koeppen H, Lu S, Hsu C-H (2020). Abstract CT044: genomic correlates of clinical benefits from atezolizumab combined with bevacizumab vs. atezolizumab alone in patients with advanced hepatocellular carcinoma (HCC). Cancer Res.

[50] Sung PS, Jang JW, Lee J, Lee SK, Lee HL, Yang H (2020). Real-world outcomes of Nivolumab in patients with Unresectable Hepatocellular Carcinoma in an endemic area of Hepatitis B Virus Infection. Front Oncol.

[51] Kim HS, Hong JY, Cheon J, Kim I, Kim CG, Kang B (2020). Different organ-specific response to nivolumab to determine the survival outcome of patients with advanced hepatocellular carcinoma (aHCC). J Clin Oncol.

[52] Huang M, He M, Guo Y, Li H, Shen S, Xie Y (2020). The influence of Immune Heterogeneity on the effectiveness of Immune checkpoint inhibitors in Multifocal Hepatocellular Carcinomas. Clin Cancer Res.

[53] Yang X, Chen B, Wang Y, Wang Y, Long J, Zhang N et al. Real-world efficacy and prognostic factors of lenvatinib plus PD-1 inhibitors in 378 unresectable hepatocellular carcinoma patients. Hepatol Int. 2023:1–11. 10.1007/s12072-022-10480-y PMC9907200 36753026

[54] Lu LC, Hsu C, Shao YY, Chao Y, Yen CJ, Shih IL (2019). Differential Organ-Specific Tumor response to Immune checkpoint inhibitors in Hepatocellular Carcinoma. Liver Cancer.

[55] Kuo HY, Chiang NJ, Chuang CH, Chen CY, Wu IC, Chang TT (2020). Impact of Immune Checkpoint inhibitors with or without a combination of tyrosine kinase inhibitors on Organ-Specific Efficacy and Macrovascular Invasion in Advanced Hepatocellular Carcinoma. Oncol Res Treat.

[56] Choi WM, Lee D, Shim JH, Kim KM, Lim YS, Lee HC et al. Effectiveness and safety of Nivolumab in child-pugh B patients with Hepatocellular Carcinoma: a real-world cohort study. Cancers (Basel). 2020;12(7). 10.3390/cancers12071968 PMC7409289 32698355

[57] Pinato DJ, Kaneko T, Saeed A, Pressiani T, Kaseb A, Wang Y et al. Immunotherapy in Hepatocellular Cancer patients with mild to severe liver dysfunction: adjunctive role of the ALBI Grade. Cancers (Basel). 2020;12(7). 10.3390/cancers12071862 PMC7408648 32664319

[58] Fessas P, Kaseb A, Wang Y, Saeed A, Szafron D, Jun T et al. Post-registration experience of nivolumab in advanced hepatocellular carcinoma: an international study. J Immunother Cancer. 2020;8(2). 10.1136/jitc-2020-001033 PMC7462152 32868393

[59] Hung HC, Lee JC, Wang YC, Cheng CH, Wu TH, Lee CF et al. Response prediction in Immune checkpoint inhibitor immunotherapy for Advanced Hepatocellular Carcinoma. Cancers (Basel). 2021;13(7). 10.3390/cancers13071607 PMC8036568 33807219

[60] Pfister D, Núñez NG, Pinyol R, Govaere O, Pinter M, Szydlowska M (2021). NASH limits anti-tumour surveillance in immunotherapy-treated HCC. Nature.

[61] Scheiner B, Pomej K, Kirstein MM, Hucke F, Finkelmeier F, Waidmann O (2022). Prognosis of patients with hepatocellular carcinoma treated with immunotherapy - development and validation of the CRAFITY score. J Hepatol.

[62] Yang Y, Ouyang J, Zhou Y, Zhou J, Zhao H (2022). The CRAFITY score: a promising prognostic predictor for patients with hepatocellular carcinoma treated with tyrosine kinase inhibitor and immunotherapy combinations. J Hepatol.

[63] Feun LG, Li YY, Wu C, Wangpaichitr M, Jones PD, Richman SP (2019). Phase 2 study of pembrolizumab and circulating biomarkers to predict anticancer response in advanced, unresectable hepatocellular carcinoma. Cancer.

[64] Chen S, Huang Z, Jia W, Tao H, Zhang S, Ma J (2020). Association of the pretreatment lung Immune Prognostic Index with Survival outcomes in Advanced Hepatocellular Carcinoma patients treated with PD-1 inhibitors. J Hepatocell Carcinoma.

[65] Myojin Y, Kodama T, Sakamori R, Maesaka K, Matsumae T, Sawai Y et al. Interleukin-6 is a circulating Prognostic Biomarker for Hepatocellular Carcinoma Patients Treated with combined immunotherapy. Cancers (Basel). 2022;14(4). 10.3390/cancers14040883 PMC8870238 35205631

[66] Ueno A, Masugi Y, Yamazaki K, Komuta M, Effendi K, Tanami Y (2014). OATP1B3 expression is strongly associated with Wnt/β-catenin signalling and represents the transporter of gadoxetic acid in hepatocellular carcinoma. J Hepatol.

[67] Aoki T, Nishida N, Ueshima K, Morita M, Chishina H, Takita M (2021). Higher enhancement intrahepatic nodules on the Hepatobiliary phase of Gd-EOB-DTPA-Enhanced MRI as a poor responsive marker of Anti-PD-1/PD-L1 monotherapy for Unresectable Hepatocellular Carcinoma. Liver Cancer.

[68] Sasaki R, Nagata K, Fukushima M, Haraguchi M, Miuma S, Miyaaki H et al. Evaluating the role of Hepatobiliary Phase of Gadoxetic Acid-enhanced magnetic resonance imaging in Predicting Treatment Impact of Lenvatinib and Atezolizumab plus Bevacizumab on Unresectable Hepatocellular Carcinoma. Cancers (Basel). 2022;14(3). 10.3390/cancers14030827 PMC8834002 35159095

[69] Qayyum A, Hwang KP, Stafford J, Verma A, Maru DM, Sandesh S (2019). Immunotherapy response evaluation with magnetic resonance elastography (MRE) in advanced HCC. J Immunother Cancer.

[70] Qayyum A, Avritscher R, Aslam R, Ma J, Pagel MD, Sun J (2020). Immune checkpoint blockade (ICB) response evaluation with MRI/MR elastography (MRE) in surgical and nonsurgical patients with HCC. J Clin Oncol.

[71] Wang G, Zhang W, Chen J, Luan X, Wang Z, Wang Y (2022). Pretreatment metabolic parameters measured by (18)F-FDG PET to predict the pathological treatment response of HCC patients treated with PD-1 inhibitors and Lenvatinib as a Conversion Therapy in BCLC Stage C. Front Oncol.

[72] Wang X, Yang X, Wang J, Dong C, Ding J, Wu M (2023). Metabolic Tumor volume measured by (18)F-FDG PET/CT is Associated with the survival of Unresectable Hepatocellular Carcinoma Treated with PD-1/PD-L1 inhibitors plus molecular targeted agents. J Hepatocell Carcinoma.

[73] Ho G, Chen S, Wong YH, Yip Y, Yung WH, Leung WT (2022). <strong > choice of tyrosine kinase inhibitor (TKI) or Immune check-point inhibitor guided by dual-tracer (11 C-acetate and 18F-FDG) PET/CT improves the progression-free survival in patients with advanced or metastatic HCC</strong >. J Nucl Med.

[74] Wu M, Wang Y, Yang Q, Wang X, Yang X, Xing H (2023). Comparison of baseline (68)Ga-FAPI and (18)F-FDG PET/CT for prediction of response and clinical outcome in patients with Unresectable Hepatocellular Carcinoma Treated with PD-1 inhibitor and Lenvatinib. J Nucl Med.

[75] Shao YY, Liu TH, Hsu C, Lu LC, Shen YC, Lin ZZ et al. Early alpha-foetoprotein response associated with treatment efficacy of immune checkpoint inhibitors for advanced hepatocellular carcinoma. Liver Int. 2019. 10.1111/liv.14210 31400295

[76] Sun X, Mei J, Lin W, Yang Z, Peng W, Chen J (2021). Reductions in AFP and PIVKA-II can predict the efficiency of anti-PD-1 immunotherapy in HCC patients. BMC Cancer.

[77] Dharmapuri S, Özbek U, Lin JY, Sung M, Schwartz M, Branch AD (2020). Predictive value of neutrophil to lymphocyte ratio and platelet to lymphocyte ratio in advanced hepatocellular carcinoma patients treated with anti-PD-1 therapy. Cancer Med.

[78] Kim CG, Kim C, Yoon SE, Kim KH, Choi SJ, Kang B et al. Hyperprogressive Disease during PD-1 blockade in patients with advanced hepatocellular carcinoma. J Hepatol. 2020. 10.1016/j.jhep.2020.08.010 32810553

[79] Ng KYY, Tan SH, Tan JJE, Tay DSH, Lee AWX, Ang AJS (2022). Impact of Immune-related adverse events on efficacy of Immune checkpoint inhibitors in patients with Advanced Hepatocellular Carcinoma. Liver Cancer.

[80] Quach HT, Dewan AK, Davis EJ, Ancell KK, Fan R, Ye F (2019). Association of Anti-programmed Cell Death 1 cutaneous toxic effects with outcomes in patients with Advanced Melanoma. JAMA Oncol.

[81] Ricciuti B, Genova C, De Giglio A, Bassanelli M, Dal Bello MG, Metro G (2019). Impact of immune-related adverse events on survival in patients with advanced non-small cell Lung cancer treated with nivolumab: long-term outcomes from a multi-institutional analysis. J Cancer Res Clin Oncol.

[82] Nigro O, Pinotti G, De Galitiis F, Di Pietro FR, Giusti R, Filetti M (2020). Late immune-related adverse events in long-term responders to PD-1/PD-L1 checkpoint inhibitors: a multicentre study. Eur J Cancer.

[83] Ao H, Xin Z, Jian Z (2021). Liquid biopsy to identify biomarkers for immunotherapy in hepatocellular carcinoma. Biomark Res.

[84] Winograd P, Hou S, Court CM, Lee YT, Chen PJ, Zhu Y (2020). Hepatocellular Carcinoma-circulating Tumor cells expressing PD-L1 are prognostic and potentially Associated with response to checkpoint inhibitors. Hepatol Commun.

[85] Li J, Jiang W, Wei J, Zhang J, Cai L, Luo M (2020). Patient specific circulating Tumor DNA fingerprints to monitor treatment response across multiple tumors. J Transl Med.

[86] Hsu C-H, Lu S, Abbas A, Guan Y, Zhu AX, Aleshin A (2020). Longitudinal and personalized detection of circulating Tumor DNA (ctDNA) for monitoring efficacy of atezolizumab plus bevacizumab in patients with unresectable hepatocellular carcinoma (HCC). J Clin Oncol.

[87] Franses JW, Lim M, Burgoyne AM, Mody K, Lennerz J, Chang J (2022). Profile and predictors of Blood Tumor Mutational Burden in Advanced Hepatocellular Carcinoma. Oncologist.

[88] Yang X, Hu Y, Yang K, Wang D, Lin J, Long J et al. Cell-free DNA copy number variations predict efficacy of immune checkpoint inhibitor-based therapy in hepatobiliary cancers. J Immunother Cancer. 2021;9(5). 10.1136/jitc-2020-001942 PMC8112417 33972389

[89] Garrett WS (2015). Cancer and the microbiota. Science.

[90] Zitvogel L, Ayyoub M, Routy B, Kroemer G (2016). Microbiome and Anticancer Immunosurveillance. Cell.

[91] Chaput N, Lepage P, Coutzac C, Soularue E, Le Roux K, Monot C (2017). Baseline gut microbiota predicts clinical response and Colitis in metastatic Melanoma patients treated with ipilimumab. Ann Oncol.

[92] Zheng Y, Wang T, Tu X, Huang Y, Zhang H, Tan D (2019). Gut microbiome affects the response to anti-PD-1 immunotherapy in patients with hepatocellular carcinoma. J Immunother Cancer.

[93] Mao J, Wang D, Long J, Yang X, Lin J, Song Y et al. Gut microbiome is associated with the clinical response to anti-PD-1 based immunotherapy in hepatobiliary cancers. J Immunother Cancer. 2021;9(12). 10.1136/jitc-2021-003334 PMC8650503 34873013

[94] Lee PC, Wu CJ, Hung YW, Lee CJ, Chi CT, Lee IC et al. Gut microbiota and metabolites associate with outcomes of immune checkpoint inhibitor-treated unresectable hepatocellular carcinoma. J Immunother Cancer. 2022;10(6). 10.1136/jitc-2022-004779 PMC9226985 35738801

[95] Llovet JM, Ricci S, Mazzaferro V, Hilgard P, Gane E, Blanc JF (2008). Sorafenib in advanced hepatocellular carcinoma. N Engl J Med.

[96] Kudo M, Finn RS, Qin S, Han KH, Ikeda K, Piscaglia F (2018). Lenvatinib versus Sorafenib in first-line treatment of patients with unresectable hepatocellular carcinoma: a randomised phase 3 non-inferiority trial. Lancet.

[97] Qin S, Bi F, Gu S, Bai Y, Chen Z, Wang Z (2021). Donafenib Versus Sorafenib in First-Line treatment of unresectable or metastatic hepatocellular carcinoma: a randomized, Open-Label, parallel-controlled phase II-III trial. J Clin Oncol.

[98] Bruix J, Qin S, Merle P, Granito A, Huang YH, Bodoky G (2017). Regorafenib for patients with hepatocellular carcinoma who progressed on sorafenib treatment (RESORCE): a randomised, double-blind, placebo-controlled, phase 3 trial. Lancet.

[99] Abou-Alfa GK, Meyer T, Cheng AL, El-Khoueiry AB, Rimassa L, Ryoo BY (2018). Cabozantinib in patients with Advanced and Progressing Hepatocellular Carcinoma. N Engl J Med.

[100] Qin S, Li Q, Gu S, Chen X, Lin L, Wang Z (2021). Apatinib as second-line or later therapy in patients with advanced hepatocellular carcinoma (AHELP): a multicentre, double-blind, randomised, placebo-controlled, phase 3 trial. Lancet Gastroenterol Hepatol.

[101] Zhu AX, Kang YK, Yen CJ, Finn RS, Galle PR, Llovet JM (2019). Ramucirumab after Sorafenib in patients with advanced hepatocellular carcinoma and increased α-fetoprotein concentrations (REACH-2): a randomised, double-blind, placebo-controlled, phase 3 trial. Lancet Oncol.

[102] Yau T, Park JW, Finn RS, Cheng A-L, Mathurin P, Edeline J et al. LBA38_PRCheckMate 459: a randomized, multi-center phase III study of nivolumab (NIVO) vs sorafenib (SOR) as first-line (1L) treatment in patients (pts) with advanced hepatocellular carcinoma (aHCC). Ann Oncol. 2019;30(Supplement_5).

[103] Kudo M, Finn RS, Edeline J, Cattan S, Ogasawara S, Palmer DH (2022). Updated efficacy and safety of KEYNOTE-224: a phase II study of pembrolizumab in patients with advanced hepatocellular carcinoma previously treated with sorafenib. Eur J Cancer.

[104] Merle P, Kudo M, Edeline J, Bouattour M, Cheng A-L, Chan SL et al. Pembrolizumab as Second-Line Therapy for Advanced Hepatocellular Carcinoma: longer term Follow-Up from the phase 3 KEYNOTE-240 trial. Liver Cancer. 2023:1–12. 10.1159/000529636 PMC10601873 37901200

[105] Qin S, Chen Z, Fang W, Ren Z, Xu R, Ryoo B-Y (2022). Pembrolizumab plus best supportive care versus placebo plus best supportive care as second-line therapy in patients in Asia with advanced hepatocellular carcinoma (HCC): phase 3 KEYNOTE-394 study. J Clin Oncol.

[106] Qin S, Kudo M, Meyer T, Bai Y, Guo Y, Meng Z et al. Tislelizumab vs Sorafenib as First-Line treatment for Unresectable Hepatocellular Carcinoma: a phase 3 Randomized Clinical Trial. JAMA Oncol. 2023. 10.1001/jamaoncol.2023.4003 PMC10557031 37796513

[107] Cheng AL, Qin S, Ikeda M, Galle PR, Ducreux M, Kim TY (2022). Updated efficacy and safety data from IMbrave150: Atezolizumab plus Bevacizumab vs. sorafenib for unresectable hepatocellular carcinoma. J Hepatol.

[108] Ryoo B-Y, Hsu C-H, Li D, Burgoyne A, Cotter C, Badhrinarayanan S (2023). Results from the MORPHEUS-liver study: phase Ib/II randomized evaluation of tiragolumab (tira) in combination with atezolizumab (atezo) and bevacizumab (bev) in patients with unresectable, locally advanced or metastatic hepatocellular carcinoma (uHCC). J Clin Oncol.

[109] Jia F, Ren Z, Xu J, Shao G, Dai G, Liu B (2020). 991P sintilimab plus IBI305 as first-line treatment for advanced hepatocellular carcinoma. Ann Oncol.

[110] Ren Z, Xu J, Bai Y, Xu A, Cang S, Du C (2021). Sintilimab plus a bevacizumab biosimilar (IBI305) versus sorafenib in unresectable hepatocellular carcinoma (ORIENT-32): a randomised, open-label, phase 2–3 study. Lancet Oncol.

[111] Xu J, Shen J, Gu S, Zhang Y, Wu L, Wu J (2021). Camrelizumab in Combination with Apatinib in patients with Advanced Hepatocellular Carcinoma (RESCUE): a nonrandomized, Open-label, phase II trial. Clin Cancer Res.

[112] Qin S, Chan SL, Gu S, Bai Y, Ren Z, Lin X (2023). Camrelizumab plus Rivoceranib versus Sorafenib as first-line therapy for unresectable hepatocellular carcinoma (CARES-310): a randomised, open-label, international phase 3 study. Lancet.

[113] Finn RS, Merle MKP, Meyer T, Qin S, Ikeda M, Xu R, Edeline J, Ryoo B, Ren Z, Cheng A, Galle PR, Kaneko S, Kumada H, Wang A, Mody K, Dubrovsky L, Siegel AB (2022). Llovet. Primary results from the phase III LEAP-002 study: Lenvatinib plus Pembrolizumab versus Lenvatinib as first-line (1L) therapy for advanced hepatocellular carcinoma (aHCC). Ann Oncol.

[114] Kelley RK, Rimassa L, Cheng AL, Kaseb A, Qin S, Zhu AX (2022). Cabozantinib plus Atezolizumab versus Sorafenib for advanced hepatocellular carcinoma (COSMIC-312): a multicentre, open-label, randomised, phase 3 trial. Lancet Oncol.

[115] Xu L, Yang JCJ, Gong W, Zhang Y, Zhao H, Yan S, Jia W, Wu Z, Liu C, Song X, Ma Y, Yang X, Gao Z, Zhang N, Zheng X, Li M, Zhang X, Chen M (2022). Efficacy and safety of tislelizumab (TIS) plus lenvatinib (LEN) as first-line treatment in patients (pts) with unresectable hepatocellular carcinoma (uHCC): a single-arm, multicenter, phase II trial. Immuno-Oncology and Technology.

[116] Gao X, Xu N, Li Z, Shen L, Ji K, Zheng Z (2023). Safety and antitumour activity of cadonilimab, an anti-PD-1/CTLA-4 bispecific antibody, for patients with advanced solid tumours (COMPASSION-03): a multicentre, open-label, phase 1b/2 trial. Lancet Oncol.

