# Supplementary material for: Biomarkers and prognostic factors of PD-1/PD-L1 inhibitor-based therapy in patients with advanced hepatocellular carcinoma
Source: Biomark Res. 2024 Feb 14;12:26. doi: 10.1186/s40364-023-00535-z (PMC10865587; doi:10.1186/s40364-023-00535-z)
Supplement: Supplementary file 1 — Supplementary Material 1 [file 40364_2023_535_MOESM1_ESM.docx]

**Supplementary Table 1.** The clinical significance of the biomarkers and prognostic factors of immunotherapy in advanced hepatocellular carcinoma

| **Biomarker/prognostic factor** | **Clinical significance** | **Detection method** | **Study design** | **Regimen [reference]** |
| --- | --- | --- | --- | --- |
| **Tumor microenvironment** |  |  |  |  |
| PD-L1 expression | Serve as a companion diagnostic to guide treatment decisions for PD-1/PD-L1 inhibitor therapy. | IHC | Interventional | ICI [5, 6], [14,15, 16, 17, 18, 19],[107] |
| TILs |  |  |  |  |
| Density | Dynamic changes are related to the immunotherapy efficacy. | IHC, mIHC/IF | Interventional | Nivolumab [22]; Tremelimumab + ablation [23]; ICI [24] |
| Spatial distribution | The presence of non-tumor area RLTCC associated with longer OS | Digital pathology and artificial intelligence | Preclinical study | [25] |
| Clonal structure | The measurement of TCR clonality can serve as a biomarker for the immunotherapy response of antigen-specific T cells. | TCR sequencing | Interventional | Tremelimumab [26] |
| Activity | CYT score is a simple and highly reproducible measure reflecting T-cell activity. A high CYT score is associated with good immunotherapy efficacy. | NGS | Preclinical study | [29] |

**Supplementary Table 1 (continued)**

| **Biomarker/prognostic factor** | **Clinical significance** | **Detection method** | **Study design** | **Regimen [reference]** |
| --- | --- | --- | --- | --- |
| Other |  |  |  |  |
| Intratumoral SDCs | Stimulate T cells by cross-presenting tumor antigens. | CyTOF | Preclinical study | [30] |
| cDC1s | Uptake cancer antigens and migrate to lymph nodes where prime CD8+ T cells concentrate. | NGS, flow cytometry, IHC | Preclinical study | [31] |
| Macrophages | Intratumoral CD38+CD68+ macrophages’ density was correlated with a better prognosis. | mIHC/IF, IHC, multiplex cytokine analysis | Preclinical study | [24] |
| CAFs | Responders owned CAFs with pro-inflammatory signaling and HCC-CAF interactions. | Spatial transcriptomics | Interventional | Cabozantinib + Nivolumab [38] |
| Expression of VEGF receptor 2, Treg, myeloid inflammation, and TREM1/MDSC signatures | It is correlated with longer PFS. | NGS, RNAseq analysis | Interventional | Atezolizumab + Bevacizumab [32] |
| Tumor cell diversity | Measuring SPP1 expression could predict the functional clonality and immune response of tumor cells; Tumor cell diversity is associated with poor prognoses. | Single-cell sequencing, machine learning | Preclinical study | [34,35] |
| Tumor immune barrier | Composed of CAFs and SPP1+ macrophages; Limit the immune cells to kill tumor cells. | Spatial transcriptomics, single-cell sequencing, and mIF | Preclinical study | [37] |

**Supplementary Table 1 (continued)**

| **Biomarker/prognostic factor** | **Clinical significance** | **Detection method** | **Study design** | **Regimen [reference]** |
| --- | --- | --- | --- | --- |
| **Genomic characteristics** | | | | |
| TMB | Serve as an independent predictive biomarker for immunotherapy efficacy;  Blood TMB is significantly correlated with tissue TMB, enabling the evaluation of blood TMB as a predictor of immunotherapy efficacy and the dynamic monitoring of treatment changes. | WES | Observational | [42,87] |
| CNAs | The burden of CNAs is correlated with the molecular typing (immune class, immune intermediate class, and immune excluded class) and immunophenotyping (proliferation class and non-proliferation class) of HCC;  High broad CNAs could serve as a predictive biomarker. | NGS | Preclinical study | [43,44] |
| TP53 | Patients with TP53 mutations exhibited a shorter OS and recurrence-free survival. | NGS | Preclinical study | [45] |
| CTNNB1 | As a biomarker of immunotherapy resistance. | NGS, RNAseq | Preclinical study | [46] |
| WNT/β-catenin signal | As a biomarker of immunotherapy resistance. | NGS | Observational | ICI [47] |
| T signaling genes | Serve as a positive prognostic biomarker. | NGS | Interventional | Atezolizumab + Bevacizumab [49] |
| Notch pathway | Serve as a negative prognostic biomarker. | NGS | Interventional | Atezolizumab + Bevacizumab [49] |
| Inflammatory gene signals | Serve as a positive prognostic biomarker. | NGS | Interventional | Nivolumab [22] |

**Supplementary Table 1 (continued)**

| **Biomarker/prognostic factor** | **Clinical significance** | **Detection method** | **Study design** | **Regimen [reference]** |
| --- | --- | --- | --- | --- |
| **Tumor clinical features** | | | | |
| Tumor burden | Smaller tumor size is correlated with better prognosis;  TBS is significantly related to the treatment response. | Imaging | Observational | Nivolumab [50]; Nivolumab [51]; PD-1 inhibitors [52];  PD-1 inhibitors + Lenvatinib [53] |
| Involved organs | Lung metastases and lymph node metastases are correlated with better prognosis. | Imaging, pathology | Observational | Nivolumab [50]; Nivolumab [51]; PD-1 inhibitors + TKI [55] |
| **Pretreatment host clinical features** | | | | |
| Child-Pugh | The grading criteria for assessing liver reserve function; Child-Pugh A indicates a better prognosis. | Laboratory tests | Observational | Nivolumab [56]; |
| ALBI | An assessment of liver function; A dependent prognostic factor. | Laboratory tests | Observational | ICI [57] |
| ECOG score | Assessing the patient's physical condition; The ECOG score of 0 is associated with a better prognosis. | Scale assessment | Observational | PD-1 inhibitors + TKI [55] |
| PG-SGA | PG-SGA score < 4 indicates a greater susceptibility to disease control. | Nutrition assessment scale | Observational | Nivolumab [59] |
| HBV/HCV infection | Viral-related HCC may benefit from immunotherapy. | Laboratory tests | Meta-analysis | Nivolumab, Atezolizumab + Bevacizumab, Pembrolizumab [60] |

**Supplementary Table 1 (continued)**

| **Biomarker/prognostic factor** | **Clinical significance** | **Detection method** | **Study design** | **Regimen [reference]** |
| --- | --- | --- | --- | --- |
| CRAFITY score | It is comprised of AFP and CRP; It could help predict the DCR and OS. | Laboratory tests | Observational | PD-1/PD-L1 inhibitors [61]; PD-1 inhibitors + TKI [62] |
| Baseline TGF-β, LDH, IL-6 | Serve as an early negative prognostic biomarker. | Laboratory tests | Interventional, observational | Pembrolizumab [63]; Nivolumab [51]; Atezolizumab + Bevacizumab [65] |
| LIPI score | A prognostic tool derived from dNLR and LDH; An early predictive biomarker of benefit. | Laboratory tests | Observational | PD-1 inhibitors [64] |
| EOB-MRI | Non-invasive diagnosis of Wnt/β-catenin mutations; EOB-MRI HBP can be used as an imaging biomarker to select the subgroup of patients with poor response. | Radiology | Observational | PD-1/PD-L1 inhibitors [67]  Atezolizumab + Bevacizumab [68] |
| MRE | Non-invasively map the viscoelastic properties of soft tissues. Increased stiffness measured by MRE is associated with unsatisfactory outcomes. | Radiology | Observational | Pembrolizumab [69]; Pembrolizumab, nivolumab +/-ipilimumab [70] |
| PET/CT | Tumor malignancy identification, primary lesion detection, and tumor staging; The ^18^F-FDG PET/CT and the development of novel tracer-guided PET/CT techniques such as dual-tracer (^11^C-acetate and ^18^F-FDG) and ^68^Ga-FAPI-PET/CT have enabled non-invasive early prediction of immunotherapy efficacy. | Radiology | Observational | PD-1 inhibitors + Lenvatinib [71]; PD-1/PD-L1 inhibitors + targeted therapy [72]; Nivolumab/pembrolizumab [73]; PD-1 inhibitors + Lenvatinib [74] |

**Supplementary Table 1 (continued)**

| **Biomarker/prognostic factor** | **Clinical significance** | **Detection method** | **Study design** | **Regimen [reference]** |
| --- | --- | --- | --- | --- |
| **Post-treatment host clinical features** | | | | |
| Dynamic changes  (AFP, PIVKA-II) | Commonly used tumor biomarkers that can dynamically monitor the patient's tumor condition | Laboratory tests | Observational | ICI [75]; PD-1 inhibitors [76] |
| PLR, NLR | Lower post-treatment PLR and NLR are linked with the clinical benefit. | Laboratory tests | Observational | Nivolumab [77]; Nivolumab [78] |
| irAE | Dermatological and endocrine irAEs are correlated with favorable prognosis. | Clinical diagnosis | Observational | ICI [79] |
| **Liquid biopsy** | | | | |
| CTC | Minimally invasive, convenient, and easily repeatable.  Distinguish unique patterns of ICI response using serial samples.  Predict ICI response based on baseline characteristics.  Predict response and resistance to ICI based on longitudinal sampling. | Immunogenicity, positive enrichment (AdnaTest, MACS, MagSweeper, CellSearch system, Biocept), negative enrichment (EasySep system, Quadrupole Magnetic Separator), enrichment based on biophysical properties | Observational | Nivolumab/Pembrolizumab [84]; |
| ctDNA |  | ddPCR, BEAMing, TAm-Seq, CAPP-Seq, WGBS-Seq, WES, WGS | Observational; Interventional | ICI [85]; Atezolizumab + Bevacizumab [86] |
| cfDNA |  |  | Observational | ICI [88] |

**Supplementary Table 1 (continued)**

| **Biomarker/prognostic factor** | **Clinical significance** | **Detection method** | **Study design** | **Regimen [reference]** |
| --- | --- | --- | --- | --- |
| **Gut microbiota** | | | | |
| The diversity and composition | Species diversity and abundance in the gut microbiota can predict patients’ prognosis and irAEs.  Metabolomic analysis of the gut microbiota holds significant promise as a meaningful direction. | Metagenomic sequencing, metabolomic analysis | Observational | PD-1 inhibitors [92]; PD-1 inhibitors [93]; ICI [94] |
| The dynamic variation characteristics |  |  |  |  |
| Metabonomic |  |  |  |  |

Abbreviations: AFP, alpha-fetoprotein; ALBI, albumin-bilirubin; BEAMing, beads, emulsion, amplification, and magnetics; CAPP-Seq, cancer personalized profiling by deep sequencing; CAFs, cancer-associated fibroblasts; cDC1, conventional dendritic cell 1; cfDNA, cell-free DNA; CRP, C-reactive protein; CTC, circulating tumor cell; ctDNA, circulating tumor DNA; CNAs, copy number alterations; CYT score, Cytolytic Activity Score; CyTOF, time-of-flight mass cytometry; dNLR, derived neutrophil-lymphocyte ratio; ddPCR, droplet digital polymerase chain reaction; DCR, disease control rate; ECOG, Eastern Cooperative Oncology Group; EOB-MRI, Gd-EOB-DTPA-enhanced magnetic resonance imaging; ^68^Ga-FAPI, ^68^Ga-labeled FAP inhibitor; HBP, hepatobiliary phase; HBV, hepatitis B virus; HCC, hepatocellular carcinoma; HCV, hepatitis C virus; ICI, immune checkpoint inhibitor; IF, immunofluorescence; IHC, immunohistochemical; IL-6, interleukin-6; irAE, immune-related adverse event; LDH, lactate dehydrogenase; mIF, multiplex immunofluorescence; mIHC, multiplex immunohistochemistry; MRE, magnetic resonance elastography; NGS, next-generation sequencing; OS, overall survival; PD-1, programmed death-1; PD-L1, programmed death ligand 1; PET/CT, positron emission tomography-computed tomography; PFS, progression-free survival; PG-SGA, patient-generated subjective global assessment; PIVKA-II, abnormal prothrombin; PLR, platelet-to-lymphocyte ratio; RLTCC, ratio of lymphocyte to total cell count; RNAseq, RNA sequencing; SDC, stimulatory dendritic cell; TBS, tumor burden score; TCR, T-cell receptor; TGF-β, Transforming Growth Factor beta; TILs, tumor-infiltrating lymphocytes; Treg, regulatory T cell; TMB, tumor mutational burden; TKI, tyrosine kinase inhibitor; TAm-Seq, tagged-amplicon deep sequencing; VEGF, vascular endothelial growth factor; WES, whole exome sequencing; WGBS-Seq, whole genome bisulfite sequencing; WGS, whole genome sequencing.
